# Cellular Models and In Vitro Assays for the Screening of modulators of P-gp, MRP1 and BCRP

**DOI:** 10.3390/molecules22040600

**Published:** 2017-04-08

**Authors:** Mariline Gameiro, Renata Silva, Carolina Rocha-Pereira, Helena Carmo, Félix Carvalho, Maria de Lourdes Bastos, Fernando Remião

**Affiliations:** UCIBIO/REQUIMTE, Laboratório de Toxicologia, Departamento de Ciências Biológicas, Faculdade de Farmácia, Universidade do Porto, Rua Jorge Viterbo Ferreira, 228, 4050-313 Porto, Portugal; mariline.gameiro@gmail.com (M.G.); helenacarmo@ff.up.pt (H.C.); felixdc@ff.up.pt (F.C.); mlbastos@ff.up.pt (M.d.L.B.); remiao@ff.up.pt (F.R.)

**Keywords:** inducers, activators, ATP-binding cassette transporters, cellular models, membrane assays, cell-based assays, in vitro assays, P-glycoprotein, multidrug resistance-associated protein 1, breast cancer resistance protein

## Abstract

Adenosine triphosphate (ATP)-binding cassette (ABC) transporters are highly expressed in tumor cells, as well as in organs involved in absorption and secretion processes, mediating the ATP-dependent efflux of compounds, both endogenous substances and xenobiotics, including drugs. Their expression and activity levels are modulated by the presence of inhibitors, inducers and/or activators. In vitro, ex vivo and in vivo studies with both known and newly synthesized P-glycoprotein (P-gp) inducers and/or activators have shown the usefulness of these transport mechanisms in reducing the systemic exposure and specific tissue access of potentially harmful compounds. This article focuses on the main ABC transporters involved in multidrug resistance [P-gp, multidrug resistance-associated protein 1 (MRP1) and breast cancer resistance protein (BCRP)] expressed in tissues of toxicological relevance, such as the blood-brain barrier, cardiovascular system, liver, kidney and intestine. Moreover, it provides a review of the available cellular models, in vitro and ex vivo assays for the screening and selection of safe and specific inducers and activators of these membrane transporters. The available cellular models and in vitro assays have been proposed as high throughput and low-cost alternatives to excessive animal testing, allowing the evaluation of a large number of compounds.

## 1. Introduction

The bioavailability of a wide variety of compounds that cannot permeate the membrane by passive diffusion (e.g., endogenous substances, drugs and other xenobiotics) is determined by the balance between uptake and efflux transporters that facilitate the movement of substrates across biological membranes [1,2,3]. Both transporters are important in maintaining cellular homeostasis, as well as in the detoxification of potentially toxic substances [2,3]. Human uptake transporters are involved in the cellular uptake of important nutrients, such as glucose and amino acids [4]. Human efflux transporters are involved in protection mechanisms against the toxicity induced by the accumulation of toxic compounds, due to their ability to export compounds out of the cells [2,4,5,6]. Similar defense mechanisms against the toxicity induced by the accumulation of pesticides and environmental pollutants are associated with efflux transporters of mammals, insects and plants [7,8].

Transporters with well-defined roles in drug efficacy are divided into 2 major groups: solute carrier (SLC) transporters superfamily (molecular weight of 40–90 kDa) and adenosine triphosphate (ATP)-binding cassette (ABC) transporters superfamily (molecular weight of 140–180 kDa) [1,2,9]. These mediate the uptake and efflux of specific substrates, respectively, determining their pharmacokinetics, safety and efficacy profiles [2,10]. Under the pharmacological point of view, efflux pumps contribute to drug bioavailability reduction, leading to multidrug resistance which compromises treatment efficacy. On the opposite, under the toxicological point of view, these efflux transporters are relevant tools to take potentially harmful xenobiotics out of the cells, consequently reducing their toxicity. The fact is that, given the concept of drug pharmacokinetics, all membrane transporters, either involved in the input or in the output processes, alter the absorption of the substances and, thus, their bioavailability. Indeed, metabolism alone does not adequately account for the observed inter-individual variability in drug disposition and response. For this reason, drug transporters, located in biological membrane barriers in relevant target organs, are recognized to be importantly involved in drug absorption, distribution and excretion and, therefore, were included in the pharmacokinetics concept, actively participating in drug bioavailability determination. Thus, the current concept of pharmacokinetics is represented by the acronym ADMET, which stands for absorption, distribution, metabolism (phase I and phase II), elimination and transport (phase 0 and phase III). Metabolism primarily occurs in the liver and intestine, being responsible for xenobiotics chemical modification: phase I includes oxidation, reduction and hydrolysis reactions, while phase II implies conjugation with different endogenous substances (e.g., glucuronic acid and glutathione) in order to increase the molecule size. In general, metabolism increases both the polarity and the size of the molecules, thus making them difficult to redistribute by tissues and facilitating, in consequence, their excretion. However, there are exceptions regarding the metabolic conversion of lipophilic compounds to hydrophilic compounds such as, for example, the acetylation and methylation reactions (phase II), which increase the lipophilicity of the compounds. Furthermore, there are cases in which the metabolism occurs to activate drugs that are free of biological activity under the form they are administered (prodrugs). Also, in some cases, biotransformation may also be responsible for the toxicity of a xenobiotic due to the formation of metabolites with a higher toxicity than the parent compound. Additionally, hepatic metabolism can contribute to bioavailability reduction with the so called “first-pass hepatic effect” or “pre-systemic elimination” for drugs with oral and rectal administration. Regarding membrane transport, SLC and ABC carriers correspond, respectively, to phase 0 and phase III transport of drugs: SLC are able to uptake drugs into cells, while ABC carriers are ATP-dependent efflux pumps, thus removing the compounds out of the cells [11]. Human SLC transporters comprise more than 400 members grouped into 52 families, playing an important role in a variety of cellular functions, often working cooperatively with other protein families. Particularly, SLC carriers can control neurotransmitters concentration in neuronal synapses, being a target for several drugs [12,13]. ABC transporters behave both as defense and resistance mechanisms against a wide range of therapeutically important drugs, influencing their disposition in the body. Indeed, ABC carriers can efficiently remove out of cells both endogenous substances and a variety of structurally unrelated exogenous compounds, including drugs, playing a relevant role in physiological, pharmacological and toxicological fields. Moreover, ABC carriers act in synergy with drug-metabolizing enzymes to protect the organism from toxic compounds. In humans, ABC transporters comprise 49 proteins distributed by seven subfamilies (ABCA–G) with about 20 carriers involved in xenobiotics transport [3,14]. Several in vitro and in vivo studies have shown that the expression and activity levels of efflux transporters are modulated by the presence of inhibitors, inducers and/or activators [15,16,17,18,19,20,21,22].

ABC transporters are widely studied due to their involvement in the development of a multidrug resistance phenotype, characterized by a decrease in the intracellular concentration of anticancer drugs, preventing the successful treatment of various forms of malignant diseases [6,7,23,24,25,26]. Although P-glycoprotein (P-gp), multidrug resistance-associated proteins 1–8 (MRP1–8) and breast cancer resistance protein (BCRP) confer resistance to anticancer drugs, only P-gp, MRP1 and BCRP seem to significantly contribute to the previously mentioned multidrug resistance phenotype [2,5,23,25].

This article focuses on the main ABC transporters involved in the multidrug resistance—P-gp, MRP1 and BCRP—expressed in tissues of toxicological relevance, such as the blood-brain barrier (BBB), cardiovascular system, liver, kidney and intestine. The main characteristics of these transporters, including their tissue distribution and the compounds able to interact with them (substrates, inhibitors, inducers and activators) are compiled in Table 1. Moreover, it provides a review of the available cellular models and in vitro assays for the screening and selection of safe and specific P-gp, MRP1 and BCRP inducers and activators. A brief section is also included regarding ex vivo approaches for the screening of ABC transporters’ inducers and activators.

## 2. Overview of the ABC Transporters

Human ABC transporters superfamily represents one of the largest families of transport proteins present in living organisms, including 49 efflux transporters organized into seven subfamilies (designed A to G) based on the similarity of gene structure, domains order and sequence homology: ABCA (12 members), ABCB (11 members; including P-gp (*ABCB1* or *MDR1* gene)), ABCC (13 members; including MRP1 (*ABCC1* gene)), ABCD (four members), ABCE (one member), ABCF (three members) and ABCG (five members; including BCRP (*ABCG2* gene)) [5,6,27,28]. These transporters move specific substrates across cell membranes (plasma and intracellular organelles membranes) against concentration gradient at the cost of ATP hydrolysis [1,2,7,29]. Consequently, the substrates accumulation inside cells is limited.

**Figure 1:**
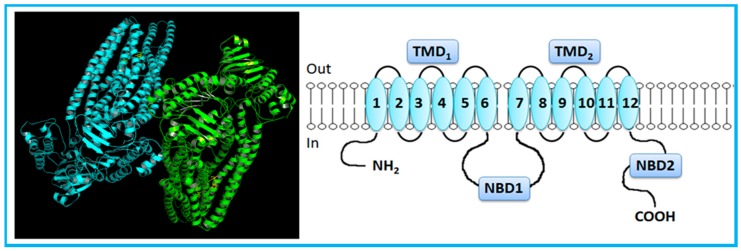
Crystal structure (Protein Data Bank (PDB) ID: 3G61) [36] and general representation of human P-glycoprotein (P-gp). P-gp, a full-transporter, contains twelve transmembrane segments, split into two halves forming transmembrane domains, each with a nucleotide-binding domain. Adapted from [3,5].

The typical topology of ABC transporters (P-gp, MRP4, MRP5, MRP8, MRP9, bile salt export pump, (BSEP)) comprises a pair of nucleotide binding domains (NBDs), located on the cytoplasmic side of the membrane, and two sets of transmembrane domains (TMDs), each containing six transmembrane-spanning α-helices (TMHs) (Figure 1) [5,8,14,30,31,32,33]. Both amine and carboxyl termini are on the cytoplasmic side of the membrane. ABC transporters with at least two TMDs and two NBDs are considered full transporters, while those with one of each domain are described as half transporters [27,32,34]. P-gp and MRP1 have a similar structure, including 12 TMHs, divided into two halves forming TMD_1_ and TMD_2_, each with a NBD (NBD_1_ and NDB_2_, respectively) [5,7,8,14,35].

**Figure 2:**
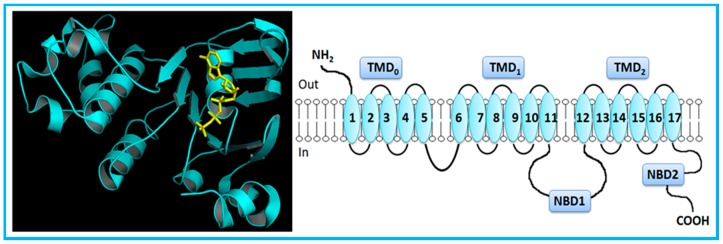
Crystal structure (PDB ID: 2CBZ) [37] and general representation of human multidrug resistance-associated protein 1 (MRP1). MRP1, a full-transporter, has three transmembrane domains, including five extra transmembrane segments toward the N-terminus, and two nucleotide-binding domains. Adapted from [5].

However, MRP1 has an additional TMD (TMD_0_) towards the N-terminus, comprising five extra TMHs (Figure 2) [5,7,14]. MRP2, MRP3, MRP6 and MRP 7 also present five extra TMHs towards the N-terminus, which is located on the extracellular side of the membrane.

**Figure 3:**
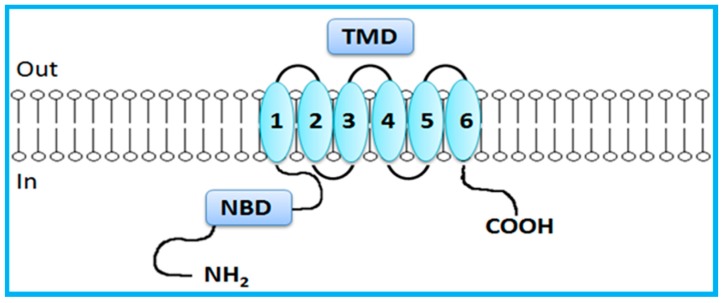
General representation of human breast cancer resistance protein (BCRP). BCRP, a half-transporter, contains only six transmembrane segments (one transmembrane domain) and one nucleotide-binding domain. Adapted from [5].

In contrast to the just mentioned full transporters, BCRP is a half transporter consisting on a single NBD and a single TMD domain, which contains 6 TMHs (Figure 3) [7,14]. Half transporters are assembled via homodimerization or heterodimerization to create a functional transport [7,23,32,34]. NBDs are directly involved in ATP binding and hydrolysis, providing energy for active transport of substrates [3]. NBDs are homologous throughout the family and have seven highly conserved motifs: the Walker A and Walker B domains, which are conserved among numerous ATP-binding proteins, and the ABC signature, the stacking aromatic and the D, Q and H loops, which are unique to ABC transporters [5,7,14,29,30,33]. TMDs form the substrate-binding site (or sites) providing the transporter specificity [14,29,30,31].

In 1997, Shapiro and Ling showed that P-gp contains at least 2 ligand-binding sites, known as H and R sites, which interact in a positively cooperative mode [38]. Later on, Shapiro et al., 1999 noted the existence of a third ligand-binding site, different from those initially proposed [39]. One year later, Martin et al., 2000 proposed the existence of 4 ligand-binding sites, classifying site I, II and III as transport sites and site IV as a regulatory site [40]. Similarly to P-gp, MRP1 and BCRP show a large number of substrates and modulators (Table 1), and probably multiple ligand-binding sites [41,42,43].

The ATP-switch model presented by Higgins and Linton is the more recent suggested transport mechanism for ABC carriers substrates [29]. According to this model, the paired NBDs switch between an ATP-dependent closed conformation and a nucleotide-free open conformation to drive the ligand translocation [30]. It is suggested a “closed NBD dimer” structure containing two ATP molecules bound at NBD dimer interface. Thus, two nucleotide binding pockets are formed with NBDs closed around. In the pocket, ATP molecules directly bind to Walker A and Walker B motifs, stacking aromatic, H and Q loops. Moreover, it is proposed that Q loop is involved in TMD-NBD contacts, which seem to be essential for substrates efflux. The transport cycle begins with the binding of substrate to the TMDs in the high-affinity opened NBD dimer conformation, increasing affinity to ATP [29,30,31]. In the second step, ATP binding induces the formation of the closed NBD dimer, which, in turn, induces a major conformational change in TMDs to start substrate transport. In the next step, ATP hydrolysis occurs and enables the conformational changes of NBD dimer dissolution to be transmitted to the TMDs. Finally, basal state of transporter is restored, after sequential release of inorganic phosphate (P_i_) and adenosine diphosphate (ADP) [29,30,31,44].

ABC transporters are highly expressed either at the apical or basolateral membranes of tumor cells, as well as in organs involved in absorption and secretion processes, especially the BBB, liver, intestine, kidney, placenta and blood-testes barrier (Table 1) [2,6,8,45,46,47,48]. Cellular localization and polarized expression of these transporters suggest that their main physiological role is to protect sensitive tissues from toxic compounds [5]. Efflux transporters reduce cellular uptake and absorption of compounds in the enterocytes, and enhance the elimination of compounds into the bile and urine, by hepatocytes and renal tubular cells, respectively. They also limit the penetration of compounds into the brain, placenta, testes, T-cells and cancer cells [1,5,7,9,45,47,48]. Noteworthy, expression and functional genetic polymorphisms of ABC transporters have been implicated in genetic diseases (e.g., cystic fibrosis, Stargardt disease, age-related macular degeneration, adrenoleukodystrophy, Tangier disease, Dubin-Johnson syndrome and progressive familial intrahepatic cholestasis), drug response and prognosis for numerous tumor types [2,8,49,50,51].

### Overview of Modulators of the ABC Transporters: Activators and Inducers

Compounds that interact with ABC transporters can act as substrates (being moved across membranes via the transporter), inhibitors (impairing the transporter-mediated efflux of other compounds), inducers (enhancing the transporter expression levels) or activators (enhancing the transporter activity), but one compound can also have overlapping modes of action [9]. All referred compounds present different properties which enables their use in distinct therapeutic applications. It should be noted that the induction and activation of the carrier are not necessarily related, since it is possible to observe an increase in the protein expression levels without a concomitant increase in its activity. Indeed, an inducer acts by promoting an upregulation in protein expression levels; and an activator binds to the protein inducing a conformational alteration which stimulates the transport of a distinct substrate bound to a different binding site [3].

Inducers and activators of the ABC transporters appear to be a useful tool in reducing the access of potentially harmful compounds to specific target tissues, due to their ability to increase the expression and activity of efflux transporters, respectively [9]. Some well-known examples of ABC carriers inducers are St. John’s wort (*Hypericum perforatum*), dexamethasone, doxorubicin, vinblastine and rifampicin for P-gp; efavirenz concerning BCRP; and sulindac for both MRP1 and MRP3 induction in human colon cancer cells (Table 1) [10,50,52,53,54]. Although the mechanism of action of P-gp inducers remains unclear, it is known that P-gp induction is regulated by nuclear factors, such as pregnane X receptor (PXR), constitutive androstane receptor (CAR), nuclear factor erythroid-derived 2-related factor (Nrf2), Y-box binding protein-1 (YB-1), nuclear factor Y (NF-Y), nuclear factor-κB (NF-κB), liver X receptor (LXR), farnesoid X receptor (FXR) and peroxisome proliferator-activated receptors α and γ (PPARα and PPARγ) [3,51,55,56,57]. The mechanism of action of P-gp activators suggests the involvement of the NBDs: (a) speed-up of the transport velocity, resulting in an increased conversion of ATP or (b) enhanced ATP affinity for the NBDs, leading to increased binding [58]; or conformational changes in the TMDs due to the binding of the compound in a specific ligand-binding site, which stimulates the substrate efflux on another ligand-binding site [20].

Several in vitro and in vivo studies have been performed to evaluate both inducers and activators applicability in the detoxification of potentially harmful compounds. Previous studies performed by our group using known P-gp inducers confirmed that P-gp induction is an effective antidotal pathway against paraquat (PQ)-induced toxicity [15,16,19,21,22,59]. In vivo studies showed that the administration of dexamethasone (100 mg/kg intraperitoneally (i.p.)) to adult male Wistar rats, 2 h after PQ exposure (25 mg/kg i.p.), resulted in a decreased PQ lung accumulation and, consequently, in an increased PQ fecal excretion. It was also observed a significant decrease in lung damage, with reduction of lipid peroxidation and carbonyl group levels, as well as normalization of myeloperoxidase activity, which resulted in a significant increase in the animals survival rate. Protection provided by dexamethasone was explained by P-gp overexpression in the cytoplasmic membrane of pneumocytes (as dexamethasone induced de novo synthesis of P-gp in these cells), causing an increased PQ elimination from lungs [59]. Two in vitro studies performed by Silva and colleagues [16,19] showed that P-gp induction by doxorubicin in human epithelial intestinal cells (Caco-2 cell line) resulted in a significant reduction of the PQ-induced cytotoxicity. In the first study, Caco-2 cells were pre-incubated with doxorubicin (0–100 µM) for 24 h, followed by PQ addition (0–5000 µM), and the cytotoxicity evaluation after 24 h of contact with the herbicide [16]. In the second study, and aiming to mimic a real-life intoxication scenario, 6 h after the initial exposure of Caco-2 cells to PQ (0–5000 µM) doxorubicin (0–100 µM) was added, the proposed antidote, and the cytotoxicity evaluated 24 h after exposure to the herbicide [19]. Using the same in vitro model, hypericin, one of the major active compounds of St. John’s wort, was described to increase both P-gp expression and activity [60]. In fact, hypericin (up to 10 μM) showed protective effects against PQ cytotoxicity under several experimental designs used (pre-incubation, co-incubation with PQ or 6 h after PQ incubation), which were completely abolished by UIC2-mediated P-gp inhibition. Additionally, Arias and co-authors recently reported the effect of pharmacological concentrations of both ethynylestradiol (EE, 0.05 pM to 5 nM) and genistein (GNT) associated with soy ingestion (0.1–10 μM) on the expression and activity of MRP2, P-gp and BCRP transporters, using the Caco-2 cell model [61]. It was clearly demonstrated an effective protection against PQ-induced cytotoxicity in cells treated with either 5 pM EE or 1 μM GNT for 48 h, when compared to control cells. Furthermore, in vitro studies performed by our research group showed that newly synthesized (thio)xanthonic derivatives prevent PQ cytotoxicity. Five thioxanthones (TX1–5) and five dihydroxylated xanthones (X1–5) were shown to be, in the Caco-2 cell model, P-gp inducers and activators [3,21,22]. Accordingly, all test compounds (20 µM) caused a significant increase in both P-gp expression and activity. When simultaneously incubated with PQ (0–7500 µM), the compounds significantly reduced PQ-mediated cytotoxicity (except TX1) and these protective effects were abolished upon incubation with a specific P-gp inhibitor [21,22]. Given the promising results obtained with these new compounds in the referred in vitro studies, ex vivo and in vivo experiments are being carried out in order to evaluate TX toxicity, toxicokinetics and antidotal ability in a living body.

Considering other cellular models, it was also reported that methylprednisolone (200 μg/mL) significantly reduced PQ cytotoxicity in the alveolar A549 cell line, an effect attributed to P-gp induction caused by the synthetic corticosteroid [62]. Moreover, other studies from our group have identified novel inducers and activators of P-gp with therapeutic potential. Vilas-Boas et al. (2013) studied the ability of both rifampicin and 3 newly synthetized rifampicin derivatives (a mono-methoxylated derivative, a peracetylated derivative and a reduced derivative) to modulate P-gp expression and activity. A model of rat’s BBB, the RBE4 cell line, was exposed to these compounds (0–50 µM). According to the results obtained, rifampicin causes a significant increase in P-gp expression at 72 h of exposure. The reduced derivative (RedRif) leads to an increase in both P-gp expression and activity at 24 h and 72 h. Pre-exposure and simultaneous exposure to the RedRif (10 µM) and PQ (0.5–50 mM) during 24, 48 and 72 h protected RBE4 cells against PQ-induced toxicity, suggesting the involvement of P-gp expression and activation mechanisms [20].

Recently, new perspectives for clinical application of ABC inducers were presented. Very importantly, Haslam and colleagues [63,64] suggested a therapeutic protection approach for chemotherapy-induced alopecia based on P-gp upregulation in hair follicle, which may reduce or prevent permanent hair loss following chemotherapy. It is proposed that ABC transporters upregulation and activation may protect melanocytes precursors in the hair follicle bulge, reducing, in consequence, the graying impact of chemotherapy [63].

In addition to the ability for carrying exogenous substrates, ABC transporters can also carry endogenous substances, such as cholesterol. This can play a role in some cholesterol-mediated pathologies in which lipids are accumulated inside cells [65]. Furthermore, several lines of research have been pointing the involvement of ABC transporters, mainly P-gp, in distinct neurodegenerative diseases, such as Alzheimer, Parkinson and epilepsy [66,67]. Because of the involvement of ABC transporters in both physiological and pathophysiological processes, there is much interest in modulating their efflux function. Alzheimer’s disease (AD) is the most common form of aged-related dementia, being characterized by the aberrant polymerization and deposition of specific misfolded proteins within the brain, particularly the insoluble and neurotoxic amyloid-β peptide (AB), which is the main component of amyloid plaque in the brain and the major pathological hallmark of AD [68,69]. AB can be transported bidirectionally across the BBB (Zlokovic 2008). Thereby, it was proposed that AB accumulation in the brain and cerebral vessels may result from 1) brain over-production, 2) aberrant clearance/transport across the BBB and 3) increased uptake from the circulation [70]. Particularly, it became clear that insufficient ABC transporter-mediated AB export, at the BBB, is involved in Alzheimer’s disease initiation and progression [71,72]. It has been shown that AB accumulates during aging due to a disturbance in its clearance from the brain, rather than the increase in its production. Moreover, despite distinct clearance pathways have been identified, the AB active efflux across the BBB seems to be the most important one [73]. P-gp, as a major efflux pump at the BBB, seems to be involved in the AB brain-to-blood transport, which could constitute an important mechanism in the pathogenesis and therapy of AD [74,75]. Thereby, it is proposed that the reduction in AB elimination from the brain into the blood can contribute to AD pathogenesis. In this context, it is known that lower levels of expression and transport activity of P-gp are correlated with higher levels of AB accumulation in the brains of older humans, as revealed by Vogelgesang and co-authors who observed that the deposition of Alzheimer’s AB is inversely correlated with P-gp expression in the brains of elderly nondemented humans [76]. Also, P-gp activity is decreased in brain regions important for memory formation in AD patients and, in a transgenic mouse model of the disease, P-gp protein level is reduced, and restoring the expression of P-gp decreases AB accumulation [68]. Cirrito and colleagues observed an increase in AB levels in the brain of P-gp-knockout mice which, according to the authors, was due to the absence of P-gp-mediated efflux when compared with wild-type mice [77]. Other researchers showed that both *Mdr1a*-knockout mice and a strain resulted from crossing *Mdr1a*-knockout mice with Tg2576 amyloid precursor protein (APP) transgenic mice (a line that routinely accumulates AB in the brain) accumulate AB in a greater extent than their respective controls [68]. Recently, it was reported a decrease in P-gp function in brains from patients with AD, as shown by positron emission tomography [78]. Consequently, the reduction/loss of P-gp expression in brain capillaries endothelium may underlie Alzheimer’s pathology. Taken together these data, the upregulation of P-gp at BBB may constitute a valid therapeutic approach for AD patients, as proposed by Abuznait and colleagues [79]. Additionally, other ABC transporters seem to be involved in AD pathogenesis. Indeed, both P-gp and BCRP are express at BBB and can share substrates, as it is the case of AB. Xiong and co-authors (2009) showed an increase in the expression pattern of BCRP in the cerebral vessels of both AD and cerebral amyloid angiopathy patients, as well as in transgenic AD mice models [80]. By using optical imaging in vivo, the authors also showed, after intravenous administration of labeled AB peptides, a significantly greater accumulation of those peptides in the brains of *Abcg2*-knockout mice comparing to the wild-type animals. This observation led the authors to purpose the upregulation of BCRP as a biomarker of amyloid angiopathy in AD patients [80]. The clearance of AB from the brain involves not only the BBB but also the blood-cerebrospinal fluid barrier (BCSFB), both expressing multiple receptors and transporters. Little is known about the role of BCSFB in AB clearance, although the choroid plexus epithelial cells, which constitute the functional component of BCSFB, express both P-gp and MRP1 [81,82]. The involvement of the mentioned ABC proteins in the above-mentioned pathology processes raises the hypothesis that these transporters could constitute potential drug targets for the treatment of distinct neurodegenerative disturbances. It has been already observed, in a clinical trial, that rifampin, a drug known to induce P-gp expression, can improve cognitive ability of patients with mild to moderate AD [83]. For this reason, the use of inducers and/or activators of ABC carriers can constitute a valid strategy to reduce AB accumulation in the brain and, therefore, to avoid the progression of neurodegenerative diseases. Because of that ever-increasing relevance of ABC transporters in multiple disease states, many efforts have been taken in order to develop and identify new putative inducers and/or activators of these protein carriers. One recent example is given by Manda and colleagues, who identified a marine-derived alkaloid as a potent P-gp inducer, establishing its structure-activity relationship [84]. Particularly, as fascaplysin induces P-gp, inhibits acetylcholinesterase and reveals a good safety profile, the authors consider it a promising anti-Alzheimer agent [84]. Other natural and synthetic compounds have been tested for P-gp induction purposes, since P-gp induction is one of the recently targeted strategy to increase AB clearance from Alzheimer brains. Phenyl benzenesulfonamides have shown ability to induce P-gp in human adenocarcinoma LS-180 cells, with an excellent therapeutic window [85]. A new compound, MC80, was shown to act as a P-gp inducer both in vitro, ex vivo and in vivo [86]. Similarly, Contino and colleagues, aiming at developing compounds able to up-regulate P-gp expression in order to reach a detoxification effect of the central nervous system (CNS) caused by AB accumulation, used an ex vivo model and identified a new benzopyrane derivative as a P-gp inducing agent [87]. Parkinson’s disease (PD) is the second most frequent neurodegenerative disorder [88]. For the same reason as that referred for AD, ABC carriers seem to be involved in PD pathophysiology since they have an important role in transporting out of the brain α-synuclein, one of the dominant proteins found in Lewy Bodies, a pathological hallmark of Lewy body disorders, including PD [89]. Particularly, several studies were carried out in order to evaluate the putative implications of polymorphisms in ABC genes in PD [90,91,92]. From these, P-gp have been receiving particular attention and a number of single-nucleotide polymorphisms in *MDR1*, the gene encoding P-gp, have been studied [93]. However, in many cases, the reported effects of these polymorphisms have been conflicting. A recently published meta-analysis conducted by Huang and colleagues suggested that ABCB11236C/T variants might play a role in the risk of PD, whereas ABCB13435C/T polymorphisms might not contribute to PD susceptibility [88]. On the other hand, a study showed that LRP1, but not P-gp, may be involved in α-synuclein efflux across BBB [89]. However, significantly greater expressions of P-gp and BCRP were detected in PD rat cerebral microvessel endothelial cells when comparing to the physiological model, and the efflux of a novel anti-PD candidate agent was exclusively attributed to P-gp [94]. Globally, the current knowledge supports the idea that P-gp is downregulated at BBB of AD and PD patients and that restoration of P-gp expression levels and/or the increase in P-gp activity by inducers and activators, respectively, could meet a potential application in the therapy of these neurological disorders. As such, pharmacological modulation of ABC transporters, namely their induction and/or activation, is of particular importance since it can be proposed as a novel therapeutic approach (e.g., prevention of chemotherapy-induced alopecia and hair pigmentation alteration, detoxification after poisoning, hypercholesterolaemia-mediated hypertrichosis, neurodegenerative disorders). By contrast, it is described an overexpression of P-gp in epileptic brain tissues, fact that would be responsible for the greater efflux of anticonvulsivant drugs, which contributes to antiseizure drug resistance [95,96]. Indeed, studies show that BBB is altered in animal models of epilepsy and in epileptic patients and P-gp is overexpressed in both in vivo and ex vivo conditions [96]. Yu and colleagues, using a rat model of epilepsy, developed a nanoparticle-infrared-Pepstatin A-based methodology to detect, by both magnetic resonance and optical imaging, P-gp in rat brains. The authors suggest this methodology as an useful tool for both the understanding of the mechanisms underlying neurological disorders and the use of P-gp-targeted therapies [97].

## 3. Study Models for ABC Transporters

According to in vivo and in vitro results obtained, inducers and activators of the ABC transporters can represent an important protection tool against xenobiotic-induced toxicity and an antidotal pathway to be explored [3,15,16,19,20,21,22]. Available cellular models and in vitro assays for the initial screening and selection of safe and specific inducers and activators of P-gp, MRP1 and BCRP have been proposed as high throughput and low-cost alternatives to excessive animal testing. In vitro methods are less expensive and less time-consuming, allowing the evaluation of a large number of compounds with a potential capacity of induction and/or activation [98]. In the following sections in vitro study models are presented.

### 3.1. Cellular Models

#### 3.1.1. Blood-Brain Barrier

The BBB is a physical and enzymatic barrier that separates the CNS from the systemic vascular environment, shielding the CNS from exposure to circulating potentially harmful compounds. In consequence, BBB regulates and protects CNS microenvironment [7,50,99,100,101].

BBB is composed of a monolayer of brain capillary endothelial cells characterized by polarized nature (apical and basal membranes, where transporters, including efflux systems such as P-gp and MRPs, are asymmetrically distributed), the paucity of both pinocytotic vesicles and fenestrations and the presence of tight junctions, metabolizing enzymes (including cytochrome P450 hemoproteins and UDP-glucuronosyltransferases) and SLC and ABC transporters [7,50,99,100,102,103]. Tight junctions form a continuous impermeable cellular barrier, preventing the entrance of large and hydrophilic compounds into the brain. Small and lipophilic molecules gain access to the brain by passive diffusion or active transport [7,50,99,100,101,103].

**Figure 4:**
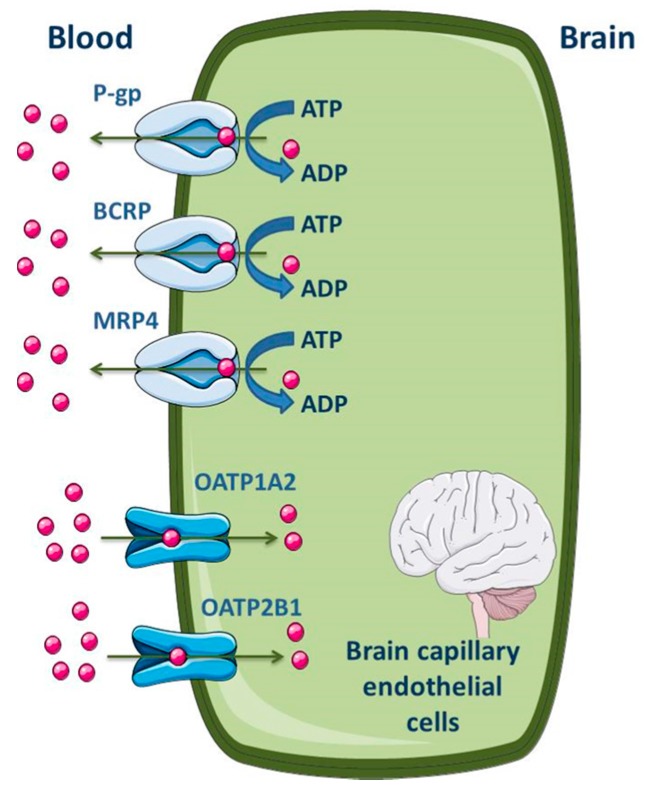
Schematic overview of the main drug transporters expressed in brain capillary endothelial cells, as well as their localization. ADP, Adenosine 5′-diphosphate; BCRP, Breast cancer resistance protein; MRP, Multidrug resistance protein; OATP, Organic anion-transporting polypeptide. Adapted from [2,11].

Several studies were performed in order to establish the level of expression of ABC transporters at the BBB of different species, including humans [104,105,106]. Differences between species were found. At the cellular level, most of the published data demonstrate that P-gp, MRP1, 2, 4, 5 and 6 and BCRP are highly expressed in the apical membrane of the brain capillary endothelial cells [7,51,52,99,100,107,108,109]. As efflux transporters, particular importance is given to P-gp, MRP4 and BCRP; regarding influx, organic anion-transporting polypeptide (OATP)1A2 and 2B1 seem to be the most expressed SLC transporters in the apical membrane of BBB (Figure 4) [2,11]. ABC efflux transporters at the BBB minimize or avoid neurotoxic adverse effects of drugs that otherwise would penetrate into the brain. However, ABC efflux transporters may also limit the central distribution of drugs that are beneficial to treat CNS diseases [99].

In vitro cellular models of the BBB started to emerge in the early 1990s, presenting multiple advantages and being complementary to in vivo studies. Cell-based BBB models can be established with any type of cell source (human, animal, or cell line derivative), including the availability of BBB endothelial cells and astrocytes freshly isolated from human brain tissue, fact that allows a considerable degree of reproducibility, both in physiological and pathological scenarios [110].

One of the most extensively characterized immortalized human cell line is the human brain endothelial capillary cell line (hCMEC/D3), a promising in vitro human BBB model for drug transport studies [111,112]. hCMEC/D3 cell line was developed by immortalization of the primary human brain capillary endothelial cells through co-expression of the human telomerase reverse transcriptase (hTERT) and Simian Vacuolating Virus 40 (SV40) large T antigen, via a lentiviral vector system [113]. The hCMEC/D3 cell line retains most of the morphological and functional characteristics of the brain endothelial cells (tight junctions, metabolizing enzymes, receptors and transporters), even in monoculture and without glial cells [108,111,113,114,115]. The hCMEC/D3 cell line maintains a stable growth and a normal BBB phenotype, including specific endothelial markers [cluster of differentiation (CD)31, CD34, CD40, CD105 and CD144, endoglin and vascular endothelial-cadherin and von Willebrand factor], at least until the 35th passage [113,115]. Weksler and colleagues (2005) identified the expression of messenger RNAs (mRNAs) coding for ABC transporters, namely P-gp, MRP1 and BCRP, and their functional activity in hCMEC/D3 cells [113]. Dauchy et al. (2009) confirmed the expression of P-gp, MRP1, MRP3–5 and BCRP genes in hCMEC/D3 cells [116]. Ohtsuki et al. (2013) performed a quantitative targeted absolute proteomic analysis of transporters and other proteins for validation of the hCMEC/D3 cell line as a useful human BBB model. P-gp, BCRP and MRP4 expression was detected, although with distinct relative expression level patterns from those found in freshly isolated human brain microvessels [114]. Although there are other human cerebral endothelial cell lines available for research purposes (including BB19, HBEC-5i, NKIM-6, hBMEC-3, TY08, TY10), the hCMEC/D3 cell line is, to date, the most well characterized [12,22,112,117,118,119]. The hCMEC/D3 is the first stable, fully characterized, well-differentiated human brain endothelial cell line, being a suitable in vitro human BBB model for drug uptake and active transport studies, as well as to study the brain endothelium response to human pathogens and inflammatory stimuli [113,115].

There are static models and static two-dimensional models of the BBB, using endothelial cells monocultures and co-culture of endothelial cells and glia, respectively [110]. In the first case, a simple monolayer of highly specialized brain microvascular endothelial cells is used. The cells, obtained from various sources (e.g., bovine, porcine, rodent, primate, human), grow in a Transwell apparatus that is a vertical side-by-side diffusion system across a microporous semipermeable membrane, allowing traffic of nutrients and of cell-derived and exogenous substances between the luminal (vascular) and abluminal (parenchymal side) compartments. Brain vascular endothelial cells grow to confluence on the luminal surface of the membrane, immersed in a specific growth media. This BBB model, with potential for using pure cell populations, allows drug permeability testing and binding affinity. However, in order to circumvent the limitations related to this model, namely its simplicity related to the absence of physiological stimuli, a two-dimensional model containing both endothelial and glia cells was developed [110]. The addition of abluminal astrocytes, in juxtaposition to the endothelial monolayer, facilitates the formation of more stringent tight junctions and the overall expression of BBB features. In addition, the exposure to glia and induced glial-endothelial interactions increases the expression of brain endothelial marker enzymes, transporters (such as P-gp and MRPs) and tight junctions, and induces a phenotype more closely mimicking that found in vivo. Transendothelial electrical resistance (TEER), a functional parameter to monitor the quality of cells cultured on filter supports, namely the integrity of the cell monolayer, is higher in co-culture of endothelial cells and glia than in endothelial cell monocultures, indicating the formation of a more stringent and selective vascular bed [110].

In addition to the cellular and static BBB models just referred, isolated brain microvessels have been extensively used to study BBB since the 1970s [120]. They have been successfully used for the identification of mechanisms and biochemical signals that play a role in regulating BBB functions in health and disease conditions, allowing the maintenance of the structural and cellular characteristics and properties in ex vivo experimentations. As such, ABC transporters, that function as efflux pumps limiting the entry of numerous xenobiotics into the brain, have been studied in isolated brain capillaries providing reliable information on the transport processes mediated by different carriers [121,122,123]. However, due to technical and functional limitations related to the use of isolated brain microvessels, computational models, artificial membranes, and in vitro cell culture BBB models have been gaining particular relevance. One of the most used computational models is the in silico model, which, knowing the physicochemical properties of novel molecules, predicts their efficacy and bioavailability in relation to BBB permeability, considering both passive diffusion and active transport processes. Consequently, computer-assisted structure-based drug design model makes the drug development process faster, predicting drug effectiveness [110].

#### 3.1.2. Cardiovascular System

The cardiac endothelial cells are characterized by expression of uptake and efflux transporters, which control the transport of a wide range of compounds, including drugs and toxins, into and out of the heart, respectively [124]. ABC transporters in the cardiovascular system, namely P-gp, MRP1, MRP3–5, MRP7 and BCRP, similar to the brain transporters, play an important protection role and were detected by different molecular biology techniques. At the end of the 1980s and in the 1990s, the expression of P-gp in several tissues was extensively studied. Studies using human heart tissues showed P-gp to be expressed in the heart, although generally at relatively low levels, when compared to tissues such as the intestine, the liver, the brain and the placenta [14]. Particularly, Meissner and colleagues observed, by immunohistochemistry and in situ hybridization, P-gp expression and location in human endothelial cells of the capillaries and arterioles of the ventricles and atria [125]. Additionally, a few years later, the authors observed the expression of BCRP in the vasculature of human heart, both in health and ischemic conditions [126]. As such, ABC transporters may provide a functional barrier between the blood and cardiomyocytes, limiting the entry of xenobiotics into the heart, namely those that are cardiotoxic, such as the anticancer drugs mitoxantrone and anthracyclines [9,14,124,126]. It should be noted that, despite distinct ABC transporters have been identified, P-gp appears to be the most relevant to cardiovascular medicine, where it modulates the efficacy and toxicity of cardioactive agents [9]. Indeed, many cardiovascular active compounds are subject to drug transport by P-gp, as it is exemplified by digoxin [127].

In addition to studies demonstrating the presence of P-gp in luminal membranes of the vascular endothelium isolated from the rat heart [128], the study performed by Estevez and colleagues demonstrated, for the first time, the presence of P-gp in primary cultures of rat heart myocytes [129].

Similarly to P-gp, other ABC carriers have been studied in the cardiovascular system. Almost all studies demonstrated the expression of MRP1 in both human and other species heart [130,131]. Other multidrug resistance proteins, such as MRP1–3 and MRP5, were identified in human heart, with higher expression in ventricular samples [132,133]. MRP5 was found to be present in cardiomyocytes and in both vascular smooth muscle and endotelial cells [132].

BCRP, an important ABC transporter mainly expressed at the BBB and placenta [134,135,136], was also studied in the heart. Its expression was found in both mouse and human hearts [126,137]. Eilers et al. studied ABC transporters gene expression by reverse transcription-polymerase chain reaction (RT-PCR) in primary human endotelial cells obtained from distinct vessels, including aorta. Here, the authors observed the presence of both P-gp and MRP1–5, proteins responsible for the efflux of the main anti-retroviral drugs. The presence of the carriers was confirmed by the suppression of the transport induced by the ABC transporters inhibitors verapamil and MK-571 (5-(3-(2-(7-Chloroquinolin-2-yl)ethenyl)phenyl)-8-dimethylcarbamyl-4,6-dithiaoctanoic acid), respectively. Human aortic endotelial cells (hAECs) expressed all the transport proteins with the following rank order: P-gp (more expressed), MRP2, MRP5, MRP1, MRP3 and MRP4 (less expressed) [138].

In another study, Higashikuni et al. showed that the human microvascular endothelial cells (hMVECs) present BCRP expression, which might be involved in tissue defense mechanisms [139]. Both cell models, hAECs and hMVECs, appear to be useful in vitro cell models for the screening and selection of potent and safe inducers and activators of the ABC transporters. The hMVECs are microvascular endothelial cells and, therefore, probably a cardiac cell model more representative of the heart transporters.

#### 3.1.3. Liver

The liver is an important tissue involved in the synthesis and secretion of bile acids, metabolism and transport of cholesterol, as well as in the metabolism and efflux of endogenous and exogenous substances [140,141]. As the major organ responsible for drug metabolism, the liver contributes to the first-pass elimination of drugs and for the plasma clearance of systemically distributed therapeutic compounds [142,143]. Therefore, together with the kidneys, the liver is an important detoxifying organ [144].

**Figure 5:**
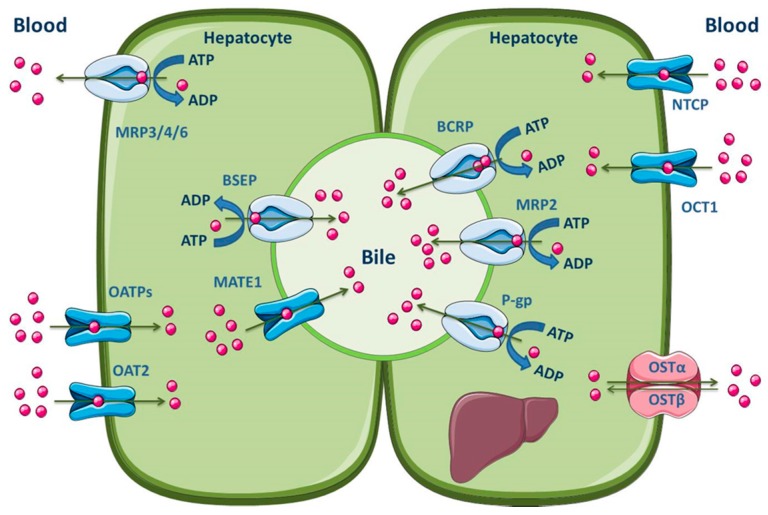
Schematic overview of the main drug transporters expressed in hepatocytes, as well as their localization. ADP, Adenosine 5′-diphosphate; ATP, Adenosine 5′-triphosphate BSEP, Bile salt export pump; MATE, Multidrug and toxin extrusion transporter; NTCP, Sodium-taurocholate co-transporting polypeptide; OAT, Organic anion transporter; OATP, Organic anion-transporting polypeptide; OCT, Organic cation transporter; OST, Organic solute and steroid transporter. Adapted from [2,11].

The hepatocytes are the most abundant cells in the liver, which represent about 80% of the liver mass [145]. These, organized in plates, have a polarized nature, apical and basolateral membranes, with different composition and functions. Hepatocytes are separated by tight junctions, which allow the vectorial transport of compounds with endogenous or exogenous origins from the blood into the bile [140,142,145,146]. The basolateral membrane is in contact with the sinusoidal blood and the canalicular membrane represents the excretory pole of hepatocytes [145]. It is known that there is a differentiated functional expression of both sinusoidal and canalicular hepatic drug transporters. Sinusoidal transporters mediate the initial step of hepatic elimination, i.e., drug uptake from the blood into the hepatocytes [147], which explains the high abundance of uptake transporters in the basolateral membrane (Figure 5). On the other hand, efflux transporters are located in both the canalicular and basolateral membranes, where they mediate excretion into bile or into the systemic circulation, respectively [140,148,149]. Major hepatic canalicular (apical) and sinusoidal (basolateral) efflux transporters are ABC proteins (Figure 5).

Those transporters located on the basolateral membrane, such as MRP1 and MRP3–6, perform the removal of endogenous compounds (organic anions and bile acids) and xenobiotics from the hepatocytes into the sinusoidal blood, for subsequent urinary elimination [150,151,152,153]. Apical efflux transporters, namely P-gp, MRP2, BCRP, multidrug and toxin extrusion 1 (MATE1) and BSEP, contribute to the biliary excretion of glutathione and glucuronide/sulfate conjugates, monoanionic bile salts and bicarbonate [2,140]. Additionally, Meyer zu Schwabedissen and Kroemer demonstrated the involvement of hepatic BCRP in the biliary excretion of some therapeutically important drugs, such as methotrexate, the 3-hydroxy-3-methylglutaryl-coenzyme A (HMG-CoA)-reductase inhibitors pitavastatin and rosuvastatin, and fluoroquinolones [154]. It should be noted, however, that the MATE1 transporter belongs to the SLC transporters (SLC47), is predominantly expressed in the canalicular membrane of hepatocytes and functions as a secondary transport system, utilizing the electrochemical gradient of cations across the membrane for substrate transport. Organic cation transporter 1 (OCT1) may function in concert with MATE1 to mediate the hepatic uptake and biliary excretion, respectively, of cationic drugs and their metabolites [155]. Other uptake transporters expressed in the hepatocyte basolateral membrane are OATP1B1, OATP1B3, OATP2B1, organic anion transporter 2 (OAT2), sodium-taurocholate co-transporting polypeptide (NTCP) and organic solute transporter α/β (OSTα/β) [2,11]. The main ABC and SLC carriers expressed in both the apical and basolateral membranes of the hepatocyte are represented in Figure 5.

Many different in vitro liver models have been employed over the years in toxicological field with the aim to predict in vivo responses. Immortalized cell lines and primary isolated liver cells are widely used in vitro models for liver toxicity testing.

HepG2 and HepaRG cell lines are common immortalized liver-derived cell lines used in laboratory protocols [156]. HepG2 cells express many liver-specific genes but the expression profile of genes involved in phase I and phase II metabolism vary between passages [157]. HepaRG cells seem to be a good alternative available for transport and drug metabolism studies, since many liver-specific functions, cytochrome P450 enzymes (CYP1A2, 2B6, 2C9, 2E1, 3A4), nuclear receptors (CAR and PXR), membrane transporters (canalicular ABC and basolateral SLC transporters) and phase II enzymes are conserved at levels comparable to those found in cultured primary human hepatocytes [158,159,160,161]. In respect to membrane carriers, HepaRG cells express substantial levels of basolateral and apical transporters also found in hepatocytes, such as OATP1B1, NTCP, OCT1 and P-gp, MRP2, BSEP, respectively. In addition, P-gp, MRP2 and BSEP were found to be inducible by nuclear receptors agonists, as already described in primary hepatocytes [150]. HepaRG cells also express aldolase B that is a specific marker of adult hepatocytes. These cells have a high proliferative capacity, being able to differentiate in both hepatocytes and biliary cells. In fact, when seeded at low density, HepaRG cells acquire an elongated undifferentiated morphology, actively divide and, after having reached confluency, form typical hepatocyte-like colonies surrounded by biliary epithelial-like cells [160]. This differentiation takes place by treatment with dimethyl sulfoxide (DMSO). Moreover, after differentiation, the expression of the different mentioned proteins remains stable for 6 weeks by treatment with DMSO [162]. However, since these cells were isolated from a grade I differentiated liver tumor of a single female patient suffering from hepatocellular carcinoma and chronic hepatitis C virus infection, their predictive value for the human population is limited [156,159].

Primary cultures of hepatocytes represent a good model for the study of hepatic drug transporters in vitro. Rodent primary hepatocyte cultures, however, may undergo the so-called de-differentiation process, which consists on changes in cell morphology, structure, polarity, gene expression and liver-specific functions (e.g., albumin production and cytochrome P450 expression). For this reason, a sandwich-based culture technique was developed [163,164]. In this system, primary hepatocytes are placed between two layers of a gelled matrix, in a sandwich configuration, retaining the in vivo-like properties. As such, cell morphology, enzymes activity, albumin production and transferrin, fibrinogen and bile salt secretion are kept close to the physiological status over a longer period of time [165]. This model is suitable for studies of hepatic drug transport, metabolism, biliary excretion and toxicity [166,167,168]. Several studies using a sandwich-cultured hepatocyte model have demonstrated enhanced morphology and viability of hepatocytes, normal levels of secretion of liver-specific transporters and CYP enzymes and organic compounds, facilitated formation of gap junctions and functional bile canalicular networks over days in culture. The main advantage of this model is the maintenance of hepatocyte’s differentiated morphology and longevity in culture [166]. On the other hand, the main sandwich-hepatocyte model disadvantage is the decrease of genes expression, responsible for many liver-specific functions, over time, although keeping itself more useful for the mechanistic studies of hepatobiliary toxicity than primary hepatocytes [169]. Sandwich-cultured human hepatocytes are considered the gold standard for the in vitro research of human hepatic transporters. However, human primary hepatocytes remain stable with time in culture, with a polarized status. Thus, monolayer-cultured human hepatocytes are also a valuable tool for the study of hepatic transporters since, contrary to that referred for rodent monolayer primary-cultured hepatocytes, the de-differentiation process is not expected to occur [170]. In fact, Schaefer and colleagues (2012) quantified sinusoidal and canalicular hepatic transporters by liquid chromatography/mass spectrometry (LC/MS) using both monolayer- and sandwich-cultured human primary hepatocytes and no major differences between them were found [171].

Due to difficulties in maintaining long-term functionality of primary hepatocytes, immortalized cells and even sandwich-hepatocytes cultures, as well as in an attempt to circumvent problems related to the small predictive value of 2D models in pharmacokinetics processes, new models have been being developed. Within these, particular attention is given to the three-dimensional models that best mimic the processes that occur in vivo. Particularly, hepatocytes have multiple apical and basolateral surfaces and, thus, their polarity is essential to safely predict, in vitro, the processes that may occur in vivo. In fact, drug uptake and diffusion in 2D systems does not accurately replicate the complexity found in a 3D multicell layer system. There are several distinct 3D hepatocyte models, which vary greatly in complexity [156,172].

Hepatocyte spheroids present a very well defined and uniform size and geometry and, although they can be differently obtained, they can replicate, in a consistent way, the biological complexities of the 3D in vivo environment, allowing a greater maintenance of functionality than that observed in the two-dimensional models. Particularly, the expression levels of enzymes of phase I metabolism are found at levels close to the physiological. In the toxicokinetics context, 3D hepatocyte spheroids allow the study of ABC proteins by quantifying drug uptake and diffusion, providing an uniform uptake by the entire surface area and avoiding complex experimental and analytical procedures [172].

Other more complex three-dimensional cultures can be used, namely systems involving porous materials, packed-bed reactors, hollow fibers and perfusion flow [156]. However, a fully functional liver culture model, where the entire in vivo dynamics can be observed, is still missing and efforts need to be carried out in order to accomplish that purpose.

#### 3.1.4. Kidney

The kidney is responsible for maintaining fluid and electrolyte homeostasis, maintaining the essential nutrients and eliminating both potentially toxic compounds and metabolic waste products from the body. These functions occur in the physiologic units of the kidney, the nephrons, composed by glomerulus and renal tubules [173,174]. The renal tubules consist of a monolayer of epithelial cells that play reabsorptive and secretory functions due to the presence of membrane transporters, which, in turn, significantly contribute to renal drug handling and for the variability in drug disposition. ABC carrier proteins are predominantly located in proximal tubules where they use the energy provided by ATP hydrolysis to move substrates across the membrane [173,174].

**Figure 6:**
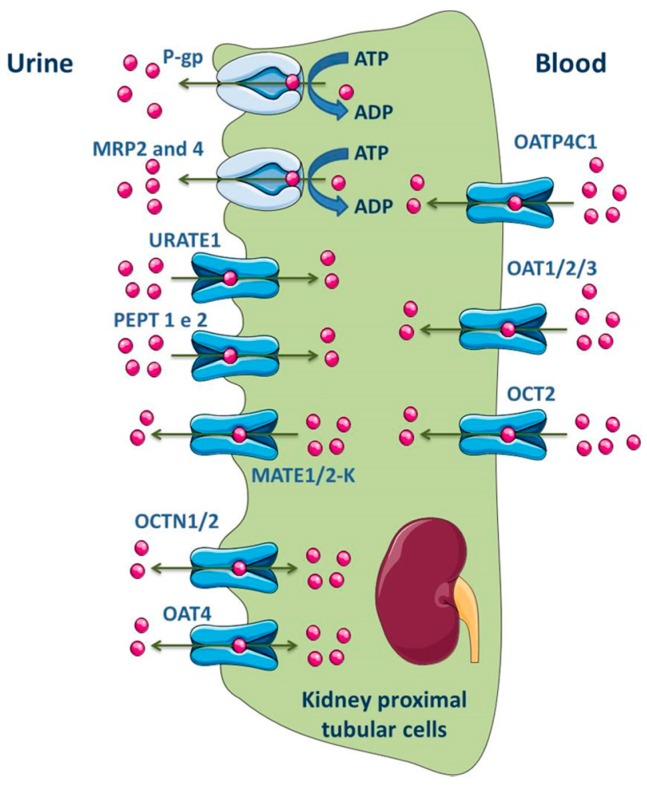
Schematic overview of main drug transporters expressed in renal epithelial cells, as well as their localization. ADP, Adenosine 5′-diphosphate; ATP, Adenosine 5′-triphosphate; OAT, Organic anion transporter; OATP, Organic anion-transporting polypeptide; OCT, Organic cation transporter; OCTN, Organic Cation/Carnitine Transporter; MRP, Multidrug resistance protein; PEPT, Peptide transporter; P-gp, P-glycoprotein; URATE, Urate transporter. Adapted from [2,11].

ABC transporters, such as MRP1, MRP3, MRP5 and MRP6, are expressed at basolateral membrane of renal tubular epithelial cells and perform the uptake of substrates into blood across this membrane. On the opposite, the efflux of substrates into glomerular filtrate is performed by transporters located at the apical membrane, namely P-gp, MRP2, MRP4 and BCRP (Figure 6) [141,174,175,176]. In fact, MRP members in proximal tubular cells function as extrusion pumps for organic anions across the apical membrane. Molecular biology techniques have shown that the renal cortical expression of MRP4 is much higher than that of MRP2 [177]. Other transporters expressed in the apical membrane of the kidney proximal tubular cells include OAT4, OCT1/2, MATE1/2-K, urate transporter 1 (URAT1) and peptide transporter 2 (PEPT2), while OATP4C1, OAT1/2/3 and OCT2 are expressed in the basolateral membrane (Figure 6) [2,11].

HK-2 (Human Kidney-2) cell line is an immortalized proximal tubule epithelial cell line derived from adult human normal kidney and retains many of the phenotypic and functional characteristics of renal proximal tubular cells in vivo [178,179,180]. To establish this cell line, a healthy kidney, unsuitable for transplantation, was used and the cortical proximal tubule segment was isolated, cultured and exposed to a recombinant virus containing the human papilloma virus (HPV) 16 *E6/E7* genes [180]. Thus, a cell clone with incorporation of HPV 16 *E6/E7* construct in the genomic DNA was designed as HK-2 and was able to continuously grow in serum free media for more than one year. At the molecular level, the products of *E6* and *E7* genes bind to the DNA regulatory proteins, resulting in facilitated cell proliferation [180,181]. Phenotypically, the HK-2 cell line has the same characteristics of normal well differentiated adult proximal tubular cells. It was shown that the HK-2 cells maintain the brush border typical enzymatic activities (acid and alkaline phosphatase, leucine aminopeptidase and gamma-glutamyl transpeptidase) [181]. Several studies were carried out using HK-2 cells to evaluate, in vitro, the renal transport processes, namely those mediated by the ABC and SLC families of transporters. Monocarboxylic acid transporter (MCT) 1–4 mRNA was shown to be present in HK-2 cells, with a predominant expression of MCT1 in the basal membrane [182]. Both OATP4C1 transporter and the efflux transporters P-gp and MRPs, but not BCRP, were also found to be present in HK-2 cells [183]. In fact, HK-2 cells retain the constitutive expression of a functional P-gp in their membranes and its activity and expression may be modulated by drugs and many commonly ingested substances [178,181,184]. In contrast to the demonstrated expression of these carriers, others were found not to be present in HK-2 cells, namely the uptake transporters OAT1, OAT3 and OCT2 [183]. According to the referred above, and despite the expression of some ABC transporters in HK-2 cells, the absence of several other transporters points to the current lack of relevant cellular models for the study of drug transport at the kidney level. Nomura and colleagues used surgically removed renal tissue and compared the ABC mRNA expression levels in human renal cell carcinomas and normal kidney tissue. In both cases, similar expression levels of MRP1–6 and P-gp were detected, with the following rank order: MRP4 (more expressed), MRP1, MRP5, P-gp and MRP3 with similar levels, MRP2 and MRP6 with similar levels (less expressed) [185].

#### 3.1.5. Intestine

**Figure 7:**
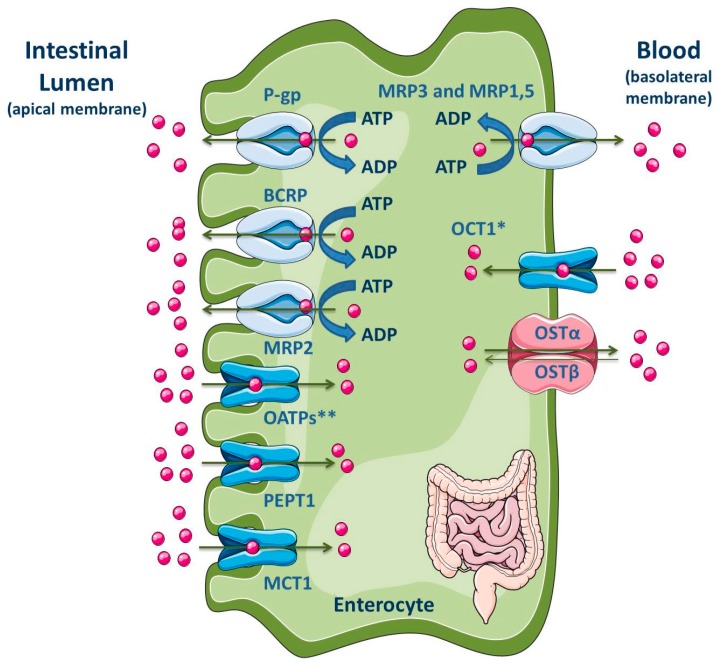
Schematic overview of main drug transporters expressed in enterocytes, as well as their localization. * Also reported in the apical membrane [189]; ** OATP2B1 also reported in the basolateral membrane of neonatal, infantile and adolescent enterocytes [190]. ADP, Adenosine 5′-diphosphate; ATP, Adenosine 5′-triphosphate; BCRP, Breast cancer resistance protein; MCT, Monocarboxylate transporter; MRP, Multidrug resistance protein; OATP, Organic anion-transporting polypeptide; OCT, Organic cation transporter; OST, Organic solute and steroid transporter; PEPT, Peptide transporter; P-gp, P-glycoprotein. Adapted from [2,11].

The intestine, in addition to the liver, is an important tissue that regulates the extent of absorption of orally administered drugs [186,187]. The majority of drug absorption occurs at the enterocytes in the small intestine, especially in the duodenum and jejunum, due to the large surface area, which is dependent on the presence of villi and microvilli [186,188]. Moreover, the intestine is known for its absorptive role due to the presence of uptake and efflux transporters, located at the apical and basolateral membranes (Figure 7), apart from the presence of cytochrome P450 3A (CYP3A4 in humans) and conjugation enzymes [186,187].

P-gp, MRP2, MRP4 and BCRP are located at the apical membrane of enterocytes, causing the drug efflux into the lumen and reducing, in consequence, the drug concentration within the enterocytes. These ABC efflux transporters are the major barrier to intestinal absorption of substrate drugs [5,9,10,186,187,188,191,192,193,194,195,196]. Moreover, the pattern of longitudinal expression of several intestinal transporters is not homogeneous along the human intestine, which may has functional implications on the preferable site of intestinal drug absorption. Additionally, their precise location (basolateral or apical) is a subject of interest and often controversial [197,198]. In fact, the expression levels of efflux transporters can vary along the small intestine. Particularly, P-gp is expressed at high levels in the ileum and colon, but it presents the lowest constitutive expression levels in the jejunum and duodenum [10,199]. BCRP is expressed in the small and large intestine but, unlike P-gp, BCRP expression does not vary significantly along the length of the small intestine [10]. P-gp, BCRP and MRP2 are located at the apical membrane, driving compounds from inside the cell back into the intestinal lumen [193,200]. Since P-gp, BCRP and MRP2 are able to bind to several structurally distinct and unrelated compounds, due to the lack of substrate specificity, they can decrease the absorption of many clinically relevant drugs, such as antibiotics, statins, HIV protease inhibitors, cardiac drugs (calcium channel blockers, digitalic), immunossupressants and anticancer agents [10].

On the contrary, MRP1 and MRP3-MRP5 are expressed at the basolateral side of enterocytes where they pump their substrates from the intracellular compartment into the systemic circulation, thereby benefiting oral bioavailability [10,193,201,202,203]. MRP1 is highly expressed in the small and large intestine, being located at the basolateral membrane of enterocytes where it functions as an absorptive carrier, avoiding the accumulation of chemicals in the enterocytes [10,204].

Some SLC proteins, responsible for drug uptake, are expressed in the apical membrane of enterocytes, such as OATPs, PEPT1, apical sodium-dependent bile acid transporter (ASBT) and MCT1; on the opposite, OSTα/β and OCT1 are expressed in the basolateral membrane (Figure 7) [2,11]. However, Han and colleagues showed the presence of OCT1 in the apical membrane of both enterocytes and Caco-2 cell monolayers [189]. Additionally, the OATP2B1 expression at the basolateral membrane of neonatal, infantile and adolescent enterocytes was recently revealed by Mooij and co-authors [190].

One of the best in vitro models of human intestinal epithelial cells available for studies of drug intestinal absorption and excretion and drug-drug interactions is the Caco-2 cell line [16,19,21,188,196,205,206]. In 1971, the Caco-2 cell line was established in culture from a human colon adenocarcinoma [207]. Caco-2 cells exhibit morphological as well as functional similarities to the human enterocytes [1]. When cultured under specific conditions, Caco-2 cells grow exponentially and, when in confluency, they undergo enterocytic differentiation, which is complete within 21 days in culture [207]. During their differentiation, they form a polarized monolayer and develop a well-defined and typical brush border with a regular microvilli on the apical surface, as well as tight cellular junctions [1,207]. These brush-border microvilli are very similar to those observed in normal small intestine and colon, with a double-leaflet plasma membrane, a core of microfilaments extending into the cytoplasm and an associated glycocalix. Caco-2 cells are indeed very similar to the small intestine enterocytes with respect to its structure and to the presence of brush-border-associated hydrolases [207,208]. Caco-2 cells have been extensively characterized and it is known that they are able to express tight junctions and very low amounts of cytochrome enzymes, making them particularly suitable as a model for examining various substrates transport properties [196]. Caco-2 cells express functional transporters involved in drugs excretion and absorption, including efflux transporters (P-gp, MRP2 and BCRP) and uptake transporters (PEPT1, OCT, OATP, ASBT and MCT), respectively [1,16,208,209]. P-gp and MRP2 expression levels seem to be similar in jejunum and Caco-2 cells, while BCRP expression levels in Caco-2 cells are low when compared with those found in the human jejunum, in vivo [210,211]. The apparent permeability coefficients measured for reference compounds across Caco-2 cells monolayers have shown good correlation with their in vivo absorption [212]. Hilgendorf et al. (2007) evaluated the mRNA expression of important drug transporters in the human intestine (jejunum and colon) and compared, by RT-PCR, with the expression levels found in Caco-2 cells. The best agreement between human tissue and the cell line was observed for the human jejunum and Caco-2 cells [192]. Intestinal peptide-associated transporter 1 (HPT1) was identified as the most abundantly expressed transporter in the intestinal mucosa. The well characterized dipeptide uptake carrier PEPT1 and the ABC efflux transporters MRP2 and BCRP were highly expressed in the jejunal tissue, associated to the following rank order: HPT1 (more expressed), PEPT1, BCRP, MRP2 and P-gp (less expressed) [192]. Our group has been showing that the Caco-2 cell line is a good in vitro model for screening and selecting potent and safe P-gp inducers/activators [16,19,21,22]. Caco-2 cells can be cultured on semi-permeable inserts, allowing the evaluation of the transport of molecules between the apical and basolateral chambers [213].

### 3.2. In Vitro Assays

Appropriate in vitro assays for transport studies can be divided in two major groups: membrane-based assays and cell-based assays.

#### 3.2.1. Membrane-Based Assays

The study of the function of the ABC efflux transporters and the identification of their substrates and inhibitors has been performed by using membranes, prepared from cells expressing ABC transporters. Similar methods can be applied in the identification of inducers and activators. Currently, there are 3 available membrane-based assays: ATPase assays, membrane vesicular transport assays and photoaffinity labeling assays [1].

Compared to cell-based assays, the membrane-based assays have several advantages, including: (1) the ability to be used to characterize the xenobiotic effects on one specific efflux transporter; (2) the ability to be easily employed in a high throughput mode; (3) the easy with which they are maintained after preparation and (4) the easy with which the assays are performed (Table 2) [1].

##### ATPase Assays

The determination of the ABC transporters ATPase activity can be performed either in isolated membranes containing the desired transporter (insect or mammalian cell membranes), or in reconstituted ABC protein preparations [32]. ATPase activity assays are commonly used in P-gp, MRPs and BCRP studies, representing a method for identification of compounds that interact with these efflux transporters [214,215].

The ATPase activity of the efflux transporters is vanadate sensitive and can be changed in the presence of substrates or modulators. These can directly interact with ABC transporters, leading to stimulation or inhibition of the formation of an intermediate state of ATPase reduction [1,214,215]. The efflux transporters can be kept in an intermediate state due to the reaction with inorganic vanadate (V_i_) and ATP. ATP hydrolysis leads to P_i_ dissociation from the transporter and is replaced by V_i_. The complex ADP·V_i_·M^2+^ (M^2+^ is a divalent cation) is formed and held in one of the NBDs of the transporter. Therefore, the ATPase activity at the active sites is completely inhibited [1].

Compounds that interact with ABC transporters can be identified as stimulators or inhibitors of their ATPase activity. Compounds that are substrates for ABC transporters-mediated transport typically stimulate their ATPase activity. The effect of the test compound on the ATPase activity of the efflux transporter is analyzed by the difference in the amount of phosphate released or, alternatively, in the remaining unmetabolized ATP, using ABC transporter expressing membranes, in the presence or absence of vanadate [1,215]. The released P_i_ levels are determined by a colorimetric reaction under mild acidic conditions, being the released P_i_ amount directly proportional to the ATPase activity of the ABC transporters. Using the other experimental approach, the quantity of unmetabolized ATP is evaluated by a luciferase-generated luminescence signal, and is inversely proportional to the ATPase activity of the ABC transporters. The assay relies on the ATP dependence of the light-generating reaction of firefly luciferase. ATP is first incubated with P-gp; then the P-gp ATPase reaction is stopped, and the remaining unmetabolized ATP is detected as a luciferase-generated luminescent signal. Therefore, a decrease in luminescence corresponds to a higher ATP consumption by the transporters, thus, the greater the decrease in luminescence signal, the higher the ATPase activity. Accordingly, samples containing compounds that stimulate the P-gp ATPase will have significantly lower signals than untreated samples. On the opposite, compounds that act as P-gp inhibitors will trigger less ATP consumption and, in consequence, the luminescence signal will be greater since the amount of unmetabolized ATP is higher. The ATP consumption that occurs in the presence of vanadate is attributed to ATPase activities of minor non-ABC transporters present in the membrane preparation. By comparing the results obtained for the basal activity and for the activity in the presence of the test compound, it can be classified into substrate, activator, inhibitor or without effect on the basal ATPase activity of the ABC transporters [1,32,215]. Furthermore, these ATPase assays can also be applied to assess kinetic parameters, such as IC_50_ for inhibitors [1].

Two different protocols can be used to study the interactions between ABC transporters and test compounds, i.e., ATPase stimulation and ATPase inhibition. In the stimulation assay, the stimulation of the basal ATPase activity of the ABC transporter is measured in the presence of the test compound. The transporter substrates significantly stimulate the basal ATPase activity. In the inhibition assay, the transporter ATPase activity is analyzed with a known substrate and a specific inhibitor. This last protocol is useful to identify inhibitory compounds and slowly transported compounds that do not change the ATPase activity [215].

Although ATPase assays allow the screening for ABC transporter substrates that can potentially act as competitive inhibitors, such as verapamil in what concerns to P-gp, resulting in the stimulation of the transporter ATPase activity, the screening for ABC transporter activators may be a tricky issue. Indeed, since this concept of a compound that immediately activates these proteins, inducing a conformational change that increases the transport of a substrate bound to another binding site, is relatively new [3], it remains unclear whether these activators are, or not, necessarily ABC transporters substrates. Therefore, two different approaches could be undertaken: the evaluation of the effect of the potential activator, alone, in the transporters ATPase activity; and the evaluation of the potential activator effect on a stimulated ATPase activity, i.e., in the presence of a typical substrate, such as verapamil in the case of P-gp. Thereby, a P-gp activator should increase the verapamil-mediated stimulation of its ATPase activity (by increasing P-gp-mediated verapamil transport); while a P-gp inhibitor should make the opposite effect. Furthermore, when evaluating the effect of the potential activator alone, it will be possible to evaluate if such compound is also a substrate, thus providing more information on the activation mechanism, namely if a co-transport of both activator and substrate might be occurring [3].

Although ATPase assays are simple, reproducible and used to detect transporter-compound interactions, these techniques are not always suitable for distinguishing among potential ABC transporter substrates and modulators, due to the presence of high intra- and inter-assay variability [1,32,216]. The ATPase assays may give false negative results for compounds, when they are studied in only one concentration, due to their low affinity and solubility. Compounds can stimulate and inhibit ABC transporters at either low or high concentrations [1].

##### Membrane Vesicular Transport Assays

The membrane vesicular transport assays are valuable tools used in the identification of substrates and modulators of the ABC transporters, such as P-gp, MRPs and BCRP. These assays can be applied in the: (a) quantification of the compound transported across the cell membrane; (b) kinetic analysis of the transported compound, including determination of the affinity constant (K_m_) and maximal velocity (V_max_); (c) study of the test compound interaction with a known substrate of the efflux transporter, to obtain the inhibitory constant (K_i_) and the half maximal inhibitory concentration (IC_50_) for inhibitors; and (d) study of the transport driving force or the requirement for the presence of co-transported molecules [1]. Therefore, these assays, although not allowing the identification of ABC transporters inducers (since the increased de novo synthesis of the proteins is needed), are useful for the identification of activators, as well as substrates and inhibitors.

The membranes used in these assays are prepared under suitable conditions and are from different sources, such as baculovirus-infected insect ovary cells, transfected or selected mammalian cell lines (from the brush border membrane of intestine, kidney and choroids plexus; hepatic sinusoidal and canalicular membranes; and luminal and abluminal membranes of the brain), transfected yeast cells and artificial membrane vesicles [1,187,216]. These contain inside-out-oriented vesicles, with both ATP- and ligand-binding sites facing the buffer outside. A rapid filtration method using glass fiber filters or nitrocellulose membranes is used to separate the vesicles from the incubation solution [1,216]. After filtration, the membrane vesicles are retained on the filter and the test compound levels embedded within the vesicles are measured by high-performance liquid chromatography (HPLC), LC/MS or LC/MS/MS. Alternatively, the compounds can be radiolabeled, fluorescent or have a fluorescent tag, being quantified the radioactivity or fluorescence retained on the filter [216]. Differences detected at level of the substrate uptake, in the presence or absence of ATP, can be attributed to transport mediated by efflux or uptake transporters, respectively [1,216].

The membrane vesicular transport assays are advantageous techniques to measure the disposition of substrates across cell membranes, including compounds with low membrane permeability and low non-specific binding [32]. The membranes can be stored at temperatures below −80 °C for many months. The membrane vesicles expressing efflux transporters are commercially available, making it possible for the routine use of these techniques [1]. However, there are also some disadvantages associated to these assays. Namely, false-negative results can be obtained in the study of compounds with medium-to-high passive permeability or highly lipophilic, due to their high nonspecific binding to the lipid membranes. Additionally, the preparation and purification protocols of the membrane vesicles are time consuming and technically complicated [1,32,187,216].

##### Photoaffinity Labeling Assays

Photoaffinity labeling assays can be divided in two major groups: detection of direct substrate/modulator binding to the ABC transporter proteins and use of a radioactively labeled ATP analog, 8-azido-ATP, applied to the analysis of the catalytic cycle and drug interactions with ABC transporters [32].

The first mentioned technique has been used in the study of the ABC transporters function, including evaluation of the binding sites, binding affinities and structural details of the substrates and modulators [1,32]. Membranes expressing ABC transporters or isolated proteins are incubated with labeled photoaffinity compounds [1]. [^3^H]azidopine (for P-gp), [^125^I]iodoaryl-azidoprazosin (for P-gp and BCRP), [^125^I]11-azidophenyl agosterol A (for P-gp and MRP), [^3^H]LTC4 (for MRP), [^125^I]iodoaryl azido-rhodamine 123 (for P-gp, MRP and BCRP) and 8-azido-[α-^32^P]ATP (for ABC proteins) are photolabeling agents frequently used, which are UV irradiated for several minutes, occurring covalent linkage of the labeled compound to the protein [1,32]. The ABC transporters radioactively labeled are solubilized and separated by gel electrophoresis. The protein labeling (drug-binding) is visualized and quantitated by autoradiography.

Another type of photolabeling assays, mentioned above and first documented for P-gp, corresponds to the use of a radioactively labeled ATP analog, 8-azido-ATP [217]. Labeled 8-azido-ATP binding, under non hydrolytic conditions, can be followed by UV-irradiation, size fractionation and autoradiography. Under hydrolytic conditions, ATP hydrolysis takes place and the binding and release of an ATP analog is too rapid to be followed. For this reason, a phosphate-mimicking transport inhibitor (e.g., vanadate, fluoroaluminate and beryllium fluoride) is used and an intermediate state complex of the ABC transporter is stabilized, carrying a hydrolyzed “trapped” nucleotide analog in the nucleotide binding pocket. The rate of the formation of this transition state can be assessed stopping the catalytic reaction by excess ATP and UV cross-linking. This formation is proportional to the rate of transport. When the substrates are efficiently transported, there is an increase in the formation of the trapped nucleotide [32].

Since both direct photoaffinity labeling and nucleotide trapping experiments are complicated techniques associated with complex protocols and are not routinely applied in the pharmaceutical industry, these techniques are important tools for studying details of the molecular mechanism. Direct photolabeling is generally not adequate for distinguishing between substrates and inhibitors [1,32]. On the other hand, ABC transporters form low-affinity interactions with a wide variety of hydrophobic compounds. The interaction sites and intensities may directly depend on the test drug and actual conformation of the transporter [32].

#### 3.2.2. Cell-Based Assays

Cell-based assays may provide more clear information about the interaction between compounds and ABC transporters, applied in the evaluation of the following kinetic parameters: K_m_ and V_max_ for substrates, and K_i_ and IC_50_ for inhibitors (Table 2).

The cytotoxicity assay is, by far, the most widely applied cell-based approach for investigating ABC transporters function. Cells expressing ABC proteins are incubated with both a known toxic substrate and the compound in study and the concentration of the test compound that leads to 50% of the maximum effect is assessed. This test compound can be an inhibitor, activator or inducer of the ABC carrier under study. For the evaluation of inducers and activators, a toxic substrate of a particular transporter may be selected and its toxicity evaluated in the presence or absence of the tested inducer/activator. Under induction/activation of a particular transporter, an increase in its expression and/or activity will result in a decreased intracellular accumulation of the toxic substrate and, therefore, in a decreased toxicity. Therefore, the evaluation of the half maximal effective concentration (EC_50_) of the selected toxic substrate should be performed (in this case, the evaluated effect is the cell death observed in the presence of different concentrations of the toxic substrate) and, under induction/activation of the specific transporter being studied, an increase in the EC_50_ value of its toxic substrate is expected to occur, as a result of a decreased toxicity. The induction/activation of the transporter may be further explored using a specific inhibitor and evaluating the disappearance of the observed protective effect of the tested inducer/activator [16,19,20,21]. Strategies to increase P-gp expression levels and/or activity may behave as effective antidotal pathways that contribute to a decrease in the intracellular accumulation of toxic compounds and cellular-based assays can be tools of particular importance for screening of new potential inducers/activators [3,22].

These assays allow a high-throughput screening of compounds due to reduced time consumption and cost, when compared, for example, with the in vivo assays, which have a high cost, are time-consuming, and have ethical restrictions. Also, the cell culture may be performed in a multi-well plate, which allows the testing of several conditions, concentrations and/or times of exposure in a single plate. However, cell-based assays are more labor and time consuming than the membrane-based assays. It is important to consider the following features: a particular cell line can express multiple transporters, although there are modified cell lines expressing one specific transporter; the culture conditions and number of cell passages may change the transporters expression levels; and the cells need to be maintained under culture conditions prior to use (Table 2) [1].

##### ABC Transporter Gene Expression

Tissue localization and changes in gene expression after cells stimulation can be monitored by Northern blot analysis, dot-blot analysis, competitive PCR, RNase protection assays or in situ hybridization. Although these methods require large RNA amounts and starting material, not allowing a rapid analysis of multiple genes and large sample numbers, they are widely accepted and reliable and can be applied to the evaluation of ABC transporters gene expression [218].

##### Real-Time Reverse Transcription-Polymerase Chain Reaction (Real-Time RT-PCR)

Real-time RT-PCR is commonly used in molecular biology for mRNA analysis, including detection and quantitation, by the use of fluorescent probes [219]. This technique is sensitive enough to enable precise and reproducible mRNA quantitation (both rare and abundant) from a single cell [219]. The evaluation of the gene expression is based on cycle threshold (Ct) values rather than end-point detection [218].

There are two main classes of chemistry compounds, i.e., DNA-binding agents (e.g., SYBR Green) and the Förster Resonance Energy Transfer (FRET) mechanism. SYBR Green is a fluorescent dye that binds to the double-stranded DNA and emits light upon excitation. The PCR product accumulation corresponds to an increase in the fluorescence intensity. Although requiring extensive optimization, this is the most economical and the easiest method. The need of optimization is related to the SYBR Green ability for binding to any double-stranded DNA during reaction, including primer-dimers and other non-specific reaction products, resulting in an overestimation of the target gene concentration. On the other hand, there are hydrolysis and hybridization FRET-based probes [219]. For the first type, each TaqMan probe comprises 15 to 25 oligonucleotides (complementary to the target sequence), a fluorescent reporter dye at the 5′ end and a quencher moiety at the 3′ end [218,219]. The proximity of the dyes, during unhybridized state, does not completely quench the fluorescence, being possible to observe a background fluorescence. During the PCR reaction, the probe anneals specifically between the primers (forward and reverse) to the desired target region of the gene. Then, the polymerase carries out the extension of the primer and replicates the template. The 5′ exonuclease activity of the polymerase cleaves the probe, releasing the reporter dye and, consequently, the intensity increases. This process is repeated in every cycle and fluorescence increases in proportion to the amount of probe cleavage. TaqMan probe does not need extensive optimization. The second FRET-based technique is based on two probes, one labeled with a fluorescent donor dye and other labeled with an acceptor dye. Once in close vicinity (3 to 5 base pairs), the donor dye emits energy that excites the acceptor dye. Consequently, there is emission of fluorescence at a different wavelength, which is monitored with a specific equipment. After each cycle, additional hybridization probes anneal, increasing the fluorescence intensity, which is measured during the exponential phase of the PCR reaction. The fluorescence intensity is proportional to the amount of input target DNA [219].

Real-time PCR allows sample processing in a multi-well plate, automatically and with high-throughput. Glyceraldehyde 3-phosphate dehydrogenase (GAPDH) is used as a reference gene for expression analysis in human tissues, but alternative reference genes can be used for other cell systems [218].

Langmann and colleagues (2003) developed a rapid, accurate and highly sensitive real-time PCR method for detection and quantification of all ABC transporters using a TaqMan probe. The following primers and probes sequences were used: *ABCB1* forward primer (5′-GTCCCAGGAGCCCATCCT-3′), *ABCB1* reverse primer (5′-CCCGGCTGTTGTCTCCATA-3′), *ABCB1* probe (5′-TTGACTGCAGCATTGCTGAGAACATTGC-3′), *ABCC1* forward primer (5′-GAAGGCCATCGGACTCTTCA-3′); *ABCC1* reverse primer (5′-CAGCGCGGACACATGGT-3′), *ABCC1* probe (5′-CTCCTTCCTCAGCATCTTCCTTTTCATGTG-3′), *ABCG2* forward primer (5′-CAGGTCTGTTGGTCAATCTCACA-3′); *ABCG2* reverse primer (5′-TCCATATCGTGGAATGCTGAAG-3′) and *ABCG2* probe (5′-CCATTGCATCTTGGCTGTCATGGCTT-3′). The method allows a rapid and complete analysis of all ABC transporters in obtained RNA samples, from twenty different human tissues. As a result, authors identified tissues involved in secretory (adrenal gland), metabolic (liver and kidney), barrier (lung, trachea and small intestine) and reproductive and tropic (placenta, uterus, prostate and testis) functions with high transcriptional activity for ABC transporters [218].

##### Flow Cytometry Assays

Flow cytometry is a rapid and specific technique that provides complete cellular analysis, being used as a tool for understanding the regulation and interaction of cell systems, mainly based in the use of fluorescent antibodies. Light emitted from these antibodies allow the identification of a wide array of cell surface and even cytoplasmic antigens [220]. Flow cytometry provides quantitative measurements of cells and other particles at a high speed, being suitable for the study of single mammalian cells in suspension by measuring their optical and fluorescence characteristics [221]. Some physical properties, such as cell size and internal complexity, can be measured by flow cytometry [220].

Fluorescent dyes may bind or intercalate with different cellular components, such as DNA or RNA. Additionally, antibodies conjugated with fluorescent dyes can bind to specific proteins on cell membranes (intact cells) or inside cells (permeabilized cells). Also, the use of fluorescent substrates, such as rhodamine 123, may be useful for the evaluation of membrane transporters activity. The labeled cells are passed by a light source and the fluorescent molecules are excited to a state of higher energy. When returning to their resting states, the fluorochromes emit light energy at higher wavelengths. The emitted fluorescence is collected using a flow cytometer, spectrally filtered and detected using photomultiplier tubes. It is possible to simultaneously measure several cell properties, using multiple fluorochromes, each one emitting light at different wavelengths, although being excited with similar wavelengths. Propidium iodide, phycoerythrin and fluorescein are commonly used dyes [220].

Flow cytometry assays can be applied to the study of ABC transporters, allowing the characterization of the interactions between drugs and ABC carriers, and usually involve the use of fluorescent transporter substrates, such as rhodamine 123 and calcein acetoxymethyl ester (calcein-AM) for P-gp [222].

Vilas-Boas and colleagues (2011) evaluated the influence of aging in P-gp expression and activity, in human lymphocytes isolated from whole blood samples of 65 healthy caucasian male donors, comparing two different methodologies. P-gp expression was analyzed using an anti-P-gp monoclonal antibody (UIC2), in the presence and absence of vinblastine. P-gp activity was studied by measuring the efflux rate of the P-gp fluorescent substrate, rhodamine 123, and by using the UIC2 shift assay. The results obtained in both studies were compared and showed a significant age-dependent increase in mean P-gp expression and no differences were found in P-gp activity. Moreover, the UIC2 shift assay proved to be more selective than the rhodamine 123 efflux assay, in the analysis of P-gp activity [223]. The researchers also used flow cytometry to study, in RBE4 cells, the putative modulatory effect of rifampicin and three rifampicin derivatives over P-gp function, using rhodamine 123 as a fluorescent substrate [20].

Recently, Silva and co-authors have been using a flow cytometry-based approach to study the ability of different compounds, such as doxorubicin, colchicine, X and TX, to modulate P-gp expression and activity, using the Caco-2 cell model. In these studies, the UIC2 monoclonal antibody conjugated with fluorescein isothiocyanate was used to study P-gp expression, and rhodamine 123 was used to evaluate P-gp activity [16,21,22,224].

Despite flow cytometry usefulness in expression and functional studies of ABC transporters in live cells, most dyes used as indicators have limited applicability as they do not simultaneously detect all types of ABC carriers [225].

##### Accumulation and Efflux Assays

Beyond flow cytometry, other accumulation and efflux assays are suitable for the screening of compounds that interfere with efflux transporters. These assays can be performed using cell suspensions, cell monolayers or membrane vesicle preparations [226].

Upon loading of the cells with lipophilic dye(s), with diffusion capacity across cell membranes, the resulting fluorescence intensity of the cell(s) will depend upon the activity of the ABC transporters [225]. The accumulation of the fluorescent substrates can be measured in the presence and absence of specific inhibitors or activators, in order to understand the effect of the transporters activity [226]. The intracellular accumulation of the dye is inversely proportional to the ABC carrier activity and can be measured by fluorescence spectrophotometry [227]. Therefore, an increased intracellular accumulation of a given substrate (higher intracellular fluorescence) can be observed in the presence of an inhibitor, while the opposite (decreased intracellular accumulation) is characteristic of an ABC transporter inducer and activator. However, the discrimination between an inducer and an activator is only related with the time of contact of such compounds with the cells. In other words, the effect of an activator, which results from an immediate change in the pump’s conformation thus increasing the efflux of a given substrate, can be observed after a short-term incubation with both activator and fluorescent substrate. On the other hand, the effect of an inducer in the pump activity requires an increased incubation period, since the de novo synthesis of the protein is needed. Moreover, to note that although an increased expression could be observed after incubation with an inducer, it will not necessarily be translated in an increased activity of a given transporter [3,16].

The efflux studies comprise the pre-load of the cells with the dye of interest. The amount of dye in the extracellular environment is measured under various conditions known to influence the transporter activity. In the presence of an inhibitor of the efflux transporter, the amount of dye expelled from the cells will be smaller than that observed for control cells. In opposition, in the presence of ABC inducers and/or activators, the amount of dye will be greater. Therefore, this method is based on the altered accumulation and/or efflux of a fluorescent substrate, such as rhodamine 123, doxorubicin, daunorubicin, or calcein-AM, that are examples of commonly used P-gp fluorescent substrates [17]. The change in the intracellular accumulation of the fluorescent compounds when co-administered with inhibitors, inducers or activators, is considered to be mainly due to their effect on the efflux pumps located in the cellular membrane, such as P-gp. It is important to notice that the analysis of the inhibition of P-gp may depend on the nature of the used substrate, since at least two binding sites, H and R, are considered to exist and inhibitors may differently interact with them. Consequently, inhibition assays may be performed with various P-gp substrates [38,228,229].

The analysis of the efflux transporters activity may be based on the evaluation of the dye accumulation, efflux or both. For example, one protocol routinely used for the evaluation of the effect of inducers or activators consists in two phases: (i) the accumulation phase, in the presence of the dye, and in which the ABC transporter activity is blocked with an inhibitor of energy production (e.g., sodium azide) and an ABC transporter inhibitor; and (ii) the efflux phase, in the absence of the dye, and where the energy-dependent ABC function is restored due to the removal of the ABC transporter inhibitor and to the addition of an energy source (e.g., medium with glucose) [16,20,223]. The first phase results in maximum substrate accumulation inside the cells. The second phase consists in restoring the normal function of the transporter, which is now able to transport the fluorescent substrate out of the cells. By analyzing the cells both after the inhibited accumulation phase and after the efflux phase, is possible to infer the amount of substrate transported by the pump. Cells with highly active transporters will display lower fluorescence intensity values due to increased efflux of the dye/substrate. For transfected cells or drug-induced cells that over-express a particular drug efflux transporter, accumulation or efflux studies can be compared to the wild-type or parental cell line that does not have as high a level of drug efflux transporter expression [225].

It is important the selection of specific inhibitors and specific fluorescent substrates. In P-gp activity studies, rhodamine 123 is frequently used as a fluorescent substrate, and cyclosporine A or PSC833 as P-gp inhibitors [16,19,20,215,222,223,224,230]. For MRP1 studies, it can be used BCECF as a substrate and probenecid or MK-571 as MRP1 inhibitors. BODIPY-prazosin, Hoechst 33342 and pheophorbide are specific BCRP fluorescent substrates, and Ko143 is a specific inhibitor [215,222,231,232].

##### Western Blotting

Western blotting or protein blotting or immunoblotting is an important technique used for the immunodetection of proteins post-electrophoresis, particularly those at low abundance [233]. Western blotting analysis is commonly performed in ABC proteins expression studies [22,234,235].

Western blotting is characterized by the following specific advantages: (a) wet membranes are flexible and of easy handling; (b) the proteins immobilized on the membrane are easily accessible to different ligands; (c) only a small amount of reagents is required for transfer analysis; (d) it is possible to obtain multiple replicas of a gel; (e) it is possible to storage transferred patterns, prior to use; (f) the same protein transfer can be used in multiple successive analysis [233].

##### Transport Assays Across Polarized Cell Monolayers

Transport assays are the most direct tool for the evaluation of transporter function and permeability of the test compound [1].

Intact cells are seeded on permeable membrane support matrix (inserts) that fit into an assay chamber, incubated at 37 °C. When cells reach confluency, they differentiate and become ready to be used in permeability studies. The two compartments are designated as apical and basolateral, denoting the membrane orientation of polarized cell layers. These two chambers are connected only through the cells monolayer and their semipermeable support. The transport differences between the basolateral-to-apical and the apical-to-basolateral compartments are easily measured. The calculated ratio is referred to as efflux ratio and for results greater than 2 the test compound is considered substrate of the active efflux transporters [1,32,187,212,226,236,237].

The experimental protocol is initiated by the addition of a solution containing the test compound to either the apical (upper chamber) or basolateral (lower chamber) compartment, for the study of the apical-to-basolateral (A-to-B) or basolateral-to-apical (B-to-A) transport, respectively [1,187,212,226,236]. On the other side is added a buffer. At desired time points, aliquots of added solution are removed from the lower chamber (for studies of A-to-B transport) or from the upper chamber (for studies of B-to-A transport). The drug cumulative amount (*Q*) present in the receiving chamber is determined as a function of the time and the steady-state flux rate (*J*) that is estimated from the obtained slope (*dQ/dt*) [1]. The apparent permeability coefficient (*P_app_*) of unidirectional flux for the test compound, calculated by the equation $P_{app}\;=\frac{J}{A\;\times \;C_{0}}$, is obtained by normalizing the flux rate *J* (mol/s) against the nominal surface area *A* and the initial compound concentration (*C_0_*, mol/mL) in the donor compartment [1,187,212]. The B/A ratio, i.e., *P_app_* value for A-to-B transport (*P_app, A-to-B_*) divided by the *P_app_* value for B-to-A transport (*P_app, B-to-A_*), or the opposite A/B ratio are easily calculated. In the presence of efflux transporters expression on the apical membrane, *P_app, A-to-B_* is smaller than *P_app, B-to-A_*. When *P_app, A-to-B_* is greater than *P_app, B-to-A_*, uptake transporter expression on the apical cell membrane occurs. These results will be contradicted if the transporter is localized on the basolateral cell membrane [1,187].

Passively diffused compounds present *P_app_* values that are independent on its concentration. The flux rate is linearly correlated with the concentration of the compound. The flux rate of actively transported compounds is saturable with increasing of its concentration. Therefore, the net transporter-mediated flux is calculated by subtracting the passive diffusion flux, and it must be estimated from the flux at 4 °C (active transporters do not function) or with specific inhibitors of the transporters. The determination of kinetic parameters, such as K_m_ and V_max_, is possible [1].

In the draft guidance from the Food and Drug Administration (FDA) on drug-drug interaction studies, transcellular transport assays are recommended as the efflux transporters functional assays, and Caco-2 and transporter-transfected Madin-Darby canine kidney strain II (MDCKII) and Lilly Laboratories cells - porcine kidney 1 LLC-PK1 (e.g., MDR1-MDCKII and MDR1-LLC-PK1) cells have been accepted as models to evaluate P-gp substrates or inhibitors. Primary cultured cells, such as primary cultured brain endothelial cells, conjunctiva and alveola epithelial cells are cell types used in these studies [1,226]. The cell type suitable for these assays must be polarized [1].

During transport assays several points should be taken into consideration, such as the selected cell line, pore size, pore density and filter material [32].

Disadvantages of the transport assays are: compounds with high passive diffusion or low permeability may result in false negatives; transfected systems and natural cell lines can present differences in the transporters expression levels or in the partitioning of substrates and, thus, can be obtained distinct K_m_ and V_max_; for cells expressing only efflux transporters, the compound transport will be limited due to the diffusion rate across basolateral membrane and, therefore, the B/A ratio may be underestimated [1].

### 3.3. Ex Vivo Assays

Many ABC carriers are constitutively expressed at the apical membrane of epithelial cells of different organs, including those that function as body barriers, such as the liver, brain, kidney and intestinal tract [238,239]. In the small intestine and colon, P-gp is one of the most important efflux proteins and may play a major contribution for several orally administered drugs bioavailability [240]. Furthermore, P-gp activation and/or induction in the intestinal mucosa may constitute a relevant approach in intoxication scenarios by decreasing toxic compounds absorption.

Ex vivo methodologies are an experimental approach where an organ or tissue is removed from the animal and placed in chambers where physiological conditions found in the living body are mimicked, namely the access to nutrients and oxygen, allowing the viability of the organ or tissue during the experimentation time.

ABC function can be accurately evaluated by using ex vivo approaches (Table 2). Indeed, the modulatory effect of different compounds over ABC carriers expressed in intestinal epithelia can be studied by using ex vivo methodologies in which everted intestinal sacs are prepared, filled with a specific ABC protein fluorescent substrate and placed in aerated buffer-containing chambers, at 37 °C. Serosal to mucosal transport of the fluorescent substrate, in the presence or absence of the putative ABC carrier modulator, is evaluated in each intestinal sac by determining the substrate concentration, by spectrofluorometry, in samples of mucosal medium, over time. Rhodamine 123 is a dye usually used as P-gp substrate [235,241,242].

### 3.4. In Silico Studies for ABC Transporters Inducers and Activators

Given the relevance of the ABC transporters in the toxicokinetics and pharmacokinetics, namely in the absorption, distribution (BBB permeation) and excretion processes, as well as their involvement in diverse pathophysiological conditions, the search for new modulators of these carrier proteins is of particular importance in both pharmacological and toxicological fields. Thereby, computational models are very valuable tools, allowing the identification of new putative ligands and, at the same time, being a relevant alternative to excessive animal testing and a preliminary approach to the in vitro and ex vivo experiments, very often expensive, laborious and time-consuming.

In silico models provide rapid and inexpensive screening platforms, and can include the development of quantitative structure-activity relationship (QSAR) models, as well as docking studies for ligand-carrier interactions prediction, and also the development of pharmacophores for ABC transporters inducers and activators [3].

Docking studies have long been used to predict the interaction of compounds with their potential targets (proteins, nucleic acids, carbohydrates and lipids). Several docking models were developed to map potential modulators of P-gp, BCRP and MRP1, thus allowing to evaluate the potential binding modes of such compounds in a given transporter [20,21,22,224,243,244,245,246,247]. Indeed, newly synthetized (thio)xanthonic derivatives demonstrated the ability to immediately increase P-gp activity after a short incubation period, an effect compatible with P-gp activation, resulting in a significant decrease in the toxicity of a P-gp substrate, PQ. The possibility of a co-transport mechanism between TXs and PQ was further supported by docking studies using a validated P-gp model [22].

However, although numerous computational models, based on QSAR analysis, pharmacophore modelling and molecular docking techniques, have been developed to predict ABC transporters substrates and inhibitors, particularly in what concerns to P-gp, the search for new inducers and activators has been mainly performed by random screening [21]. Noteworthy, and in an attempt to address this gap, pharmacophores for P-gp inducers and activators were recently developed, which can be of utmost importance, in the future, in predicting new ligands [22,224]. In fact, based on the in vitro P-gp activation ability of newly synthetized thioxanthonic derivatives [22] and on a set of known P-gp activators described in the literature, the authors developed and validated common feature pharmacophore models for P-gp activation. The best ranked pharmacophore reported was composed of three features (one hydrophobic feature, one aromatic ring, and one hydrogen bond acceptor group) and can be a very useful tool to efficiently and rapidly predict new ligands with the ability to activate P-gp.

Additionally, pharmacophore construction was also performed for P-gp inducers. Briefly, the pharmacophores were validated using known P-gp inducers and can be used to map new compounds, as it was the case of newly synthetized TXs, for which there was previous indication from data of in vitro assays about their potential to activate and induce P-gp. However, since many signalling transduction pathways can be considered in regulating the expression of a given transporter, fact that is particular evident for ABC transporters, and given the structural diversity of the compounds, finding a pharmacophore for P-gp inducers can be a challenging task. Noteworthy, by using such pharmacophores for P-gp inducers and activators, a perfect match between in silico and in vitro studies was observed [21,22], thus further reinforcing the idea that the use of such in silico strategies can help to predict the P-gp modulatory effects of new drugs that can be initially screened through these newly developed pharmacophores.

Also, in vitro data on the ability of newly synthetized dihydroxylated xanthones to activate P-gp and protect Caco-2 cells against the cytotoxicity induced by a P-gp substrate, PQ, triggered the development of a 2D QSAR model, which demonstrated that the maximal partial charge for oxygen atoms is related with the P-gp activation ability of such compounds [21]. Furthermore, a perfect match was again observed, with both the docking studies and the QSAR model being in accordance with the reported in vitro data [21].

Taken together, the in silico models disclose new possibilities in drug discovery and can be a valuable and complementary tool in the prediction of new ligands, allowing a more rational use of in vitro, ex vivo and in vivo assays.

## 4. Conclusions

In vitro and in vivo studies with inducers and activators of the ABC transporters have shown that the use of these compounds may be an effective antidotal pathway against xenobiotic-induced toxicity. The action mechanisms of both are not clear. Therefore, it is important to conduct more research involving putative inducers and activators of the ABC transporters, in order to understand: (1) their mechanism of action; (2) their specificity and (3) their toxicity in tissues with toxicological relevance. Furthermore, it is important to synthesize new compounds with specificity for ABC transporters, mainly those involved in multidrug resistance phenotype (P-gp, MRP1 and BCRP).

During the assessment of new modulators of the ABC transporters it is important to use adequate in vitro assays, high throughput and low-cost alternatives to excessive animal testing, evaluating their main effects on the expression and activity of the ABC transporters. Using only one technique or one concentration of the test compound could lead to false results. The antidotal therapeutic application of the studied compounds in vitro should be studied exposing a cell line to a toxic substrate of a particular ABC transporter in the presence of a potential inducer/activator, and calculating the xenobiotic EC_50_ value. The results should be compared with results obtained in the absence of the tested inducer/activator. For compounds that increase the ABC transporter expression and/or activity, a protective effect against the xenobiotic-induced toxicity should be observed and represented by an increase in the EC_50_ value of the used toxic substrate.

## Figures and Tables

**Table 1:** Properties of the main adenosine triphosphate (ATP)-binding cassette (ABC) transporters involved in multidrug resistance.

Protein and Gene Name	Tissue Distribution	Substrates	Inhibitors	Inducers	Activators
P-gp (*ABCB1* or *MDR1*)	Brain, liver, kidney, intestine, uterus, ovary, testes, placenta, adrenal gland, cancer cells [1,3,14]	**Anticancer drugs** (vinca alkaloids (vinblastine, vincristine, catharanthine), anthracyclines (doxorubicin, daunorubicin), taxanes (paclitaxel and docetaxel), epipodophyllotoxins (etoposide, teniposide), camptothecins (topotecan, methotrexate), anthracenes (bisantrene, mitoxantrone)) **HIV protease inhibitors** (ritonavir, saquinavir, nelfinavir, amprenavir, indinavir, maraviroc, darunavir) **Analgesics** (morphine) **Antidepressants** (amitryptiline, nortryptiline, doxepin, venlafaxine, paroxetine) **Antihistamines** (terfenadine, fexofenadine) **Histamine H2-receptor antagonists** (cimetidine) **Antidiarrheal agents** (loperamide) **Immunosuppressive agents** (sirolimus, valspodar, cyclosporin A, tacrolimus (FK506) **Antiarrhythmics** (quinidine, amiodarone, propafenone) **Antiepileptics** (phenytoin, felbamate, topiramate, carbamazepine, lamotrigine, phenobarbital, gabapentin, topiramate) **Fluorescent compounds** (calcein-AM, Hoechst 33342, rhodamine 123) **HMG-CoA reductase inhibitors** (lovastatin, simvastatin) **Antiemetics** (ondansetron, domperidone) **Antimicrobial agents** (doxycycline, erythromycin, itraconazole, ketoconazole, levofloxacin, rifampin, sparfloxacin, tetracycline, grepafloxacin), **Antihelminthics** (abamectin, ivermectin) **Tyrosine kinase inhibitors** (imatinib mesylate, gefitinib, nilotinib, tandutinib) **Cardiac glycosides** (digitoxin, digoxin, quinidine) **Calcium-channel blockers** (verapamil, nifedipine, azidopine, diltiazem, nicardipine) **Calmodulin antagonists** (trifluoperazine, chlorpromazine, trans-flupentixol) **Anti-tuberculous agent** (rifampin) **Anti-hypertensives** (debrisoquine, reserpine, propranolol, celiprolol, diltiazem, losartan, talinolol, prazosin) **Anticonvulsants** (phenobarbital, phenytoin) **Antibiotics** (actinomycind, clarithromycin, amoxicillin, erythromycin, gramicidin A, valinomycin, tetracyclines, fluoroquinolines) **Anti-gout agents** (colchicine) **Anti-tuberculous agent** (erythromycin) **Thrombin inhibitors** (dabigatran) **Steroids** (dexamethasone, hydrocortisone, corticosterone, cortisol, aldosterone, methylprednisolone) **Opioids** (loperamide, morphine, pentazocine) **Pesticides** (methylparathion, endosulfan, paraquat, cypermethrin, fenvalerate) **Nature products** (flavonoids, curcuminoids, Rhei Rhizoma extract) **Antialcoholism drug** (disulfiram) [3,5,10,14,99,102,174,191]	**1st generation inhibitors:** (verapamil, cyclosporin A, nifedipine, quinidine, quinine, amiodarone, tamoxifen detergents (cremophore EL)) **2nd generation inhibitors:** (R-verapamil, PSC 833, dexniguldipine, valspodar, elacridar, biricodar, dexverapamil, dofequine fumarate) **3rd generation inhibitors:** (ontogen (OC 144-093), zosuquidar, tariquidar, elacridar laniquidar, biricodar) **4th generation inhibitors:** surfactants (e.g., sodium dodecyl sulphate, Tween-20 and Span-80) and lipids; compounds extracted from natural origins and their derivatives (flavonoids, such as tangeretin, sinensetin, baicalein and quercetin; alkaloids, such as ellipticine ; coumarins such as cnidiadin and praeruptorin; among others); peptidomimetics (e.g., reversins) and agents combining transport inhibition with another beneficial biological activity, also called dual ligands (aminated thioxanthones, that act as dual inhibitors of cell growth and P-gp (e.g., 1-[2-(1*H*-benzimidazol-2yl)ethanamine]-4-propoxy-9*H*-thioxanthen-9-one), which also demonstrated to be a potent inhibitor of other ABC transporters, such as BCRP, MRP-1 and MRP-3). [17,99,226]	The *MDR1* gene responds to a vast diversity of internal or external chemical stimuli (e.g., drugs, cytokines, oxygen free radicals, tumor suppressor genes and heat shock) and to other environmental factors, such as X-irradiation, UV-irradiation. Some of the reported P-gp inducers comprise (listed alphabetically): Abacavir, Actinomycin D, Aflatoxin B1, Aldosterone, Ambrisentan, Amiodarone, amprenavir, m-amsacrine, apigenin, artemisinin, asiatic acid, atazanavir, atorvastatin, avermectin, beclomethasone, benzo(a)pyrene, benzo(e)pyrene, berberine, betamethasone, bilirubin, bosentan, bromocriptine, budesonide, caffeine, cadmium chloride, capsaicin, carbamazepine, catechin, celiprolol, cembratriene, *R*-cetirizine, CITCO, chlorambucil, cholate, chrysin, ciclesonide, cisplatin, clotrimazole, colchicine, corticosterone, curcuma extracts (extracts of *Curcuma longa*, *Curcuma zedoaria* and *Curcuma aromatica*), curcumin, cyanidin, cyclophosphamide, cyclosporine A, cytarabine, daidzein daunorubicin, daurunavir, depsipeptide (FR901228, FK228, NSC630176), desvenlafaxine, dexamethasone, diclofenac, digoxin, dihydroxylated xanthones (e.g., 1,2-dihydroxy-9*H*-xanthen-9-one (X2)), 1α,25-dihydroxyvitamin D3, diltiazem, dimethylformamide, 6,16α-dimethylpregnenolone, dimethylsulfoxide, docetaxel, doxorubicin, doxycycline, efavirenz, emetined, epigallocatechin-3-gallate, epirubicin, eriodictyol, erythromycin, β-estradiol, ethinylestradiol, etoposide, fenbufen, flavone, 5-fluorouracil, flutamide, fluticasone, genistein, ginkgolides a and b, hydroxyurea, hyperforin, hypericin, *Hypericum perforatum* extracts (Saint John’s wort), idarubicin, ifosfamide, indinavir, indomethacin, insulin, isosafrole, isoxanthohumol, ivermectin, lopinavir, LY191401, mangiferin, meloxicam, mepirizole, methotrexate, methylprednisolone, midazolam, mifepristone, mitoxantrone, morphine, mx2, myricetin, naringenin, nefazodone, nelfinavir, nevirapine, nicardipine, nifedipine, nimesulide, norathyriol, oleocanthal, ouabain, oxycodone, paclitaxel, parthenolide, pentylenetetrazole, phenobarbital, phenothiazine, phenytoin, phorbol 12-myristate 13-acetate, piperine, platelet-activating factor, prednisolone, 5β-pregnane-3,20-dione, pregnenolone-16α-carbonitrile, probenecid, propranolol, quercetin, quinidine, rapamycin or sirolimus, reduced rifampicin derivative (RedRif), rescinnamine, reserpine, retinoic acid, rhinacanthin-C, rifampicin, rilpivirinem, ritonavir, saquinavir, small molecule tyrosine kinase inhibitors (erlotinib, gefitinib, nilotinib, sorafenib, vandetanib), sildenafil, sodium arsenite, sodium butyrate, spironolactone, SR12813, sulindac, tacrolimus, tadalafil, tamoxifen, tangeretin, taurocholate, taxifolin, TCDD, tetrahydrocurcumin, thioxanthonic derivatives (e.g., 1-(propan-2-ylamino)-4-propoxy-9*H*-thioxanthen-9-one (TX 5)), γ-tocotrienol, topotecan, trazodone, triactyloleandomycin, trichostatin A, trimethoxybenzoylyohimbine, venlafaxine, verapamil, vinblastine, vincristine [3].	A synthetic derivative of rifampicin (a reduced derivative, RedRif) [20] Dihydroxylated xanthones: 3,4-Dihydroxy-9*H*-xanthen-9-one (X1), 1,2-Dihydroxy-9*H*-xanthen-9-one (X2), 1,3-Dihydroxy-9*H*-xanthen-9-one (X3), 2,3-dihydroxy-9*H*-xanthen-9-one (X4) and 3,6-dihydroxy-9*H*-xanthen-9-one (X5) [21] Thioxanthonic derivatives: 1-[(3-hydroxypropyl)amino]-4-propoxy-9*H*-thioxanthen-9-one (TX 1), 1-chloro-4-hydroxy-9*H*-thioxanthen-9-one (TX 2), 1-{[2-(1,3-benzodioxol-5-yl) ethyl]amino}-4-propoxy-9*H*-thioxanthen-9-one (TX 3), 1-[(2-methylpropyl) amino]-4-propoxy-9*H*-thioxanthen-9-one (TX 4) and 1-(propan-2-ylamino)-4-propoxy-9*H*-thioxanthen-9-one (TX 5) [22]
MRP1 (*ABCC1*)	Brain, kidney, lung, intestine and testis [14]	**Anticancer drugs** (vinca alkaloids (vinblastine, vincristine), anthracyclines (doxorubicin, daunorubicin), taxanes (paclitaxel), epipodophyllotoxins (etoposide, teniposide), camptothecins (topotecan, methotrexate, irinotecan)) **Glucuronide, sulfate and glutathione conjugates, and unconjugated compounds** (glucuronosylbilirubin, estradiol-17-β-d-glucuronide, etoposide-glucuronide, SN-38-glucuronide, leukotrienes C4, D4 and E4, glutathione disulfide, prostaglandin A2-SG, hydroxynonenal-SG, aflatoxin B1-epoxide-SG, cyclophosphamide-SG, doxorubicin-SG, estrone-3-sulfate, dehydroepiandrosterone-3-sulfate, sulfatolithocholyl taurine) **Antibiotics** (difloxacin, grepafloxicin) **Folates** (folic acid, l-leucovorin) **Anthracenes** (mitoxantrone) **Natural products** (curcuminoids) **Metalloids** (sodium arsenate, sodium arsenite, potassium antimonite) **Pesticides** (fenitrothion, methoxychlor) **Toxins** (aflatoxin B1) **Fluorescent compounds** (calcein, fluorescein, Fluo-3, BCECF) **HIV protease inhibitors** (indinavir, adefovir) [1,5,10,14,99,102]	Sulfinpyrazone, biricodar, probenecid, MK571, LTC4, cyclosporin A, verapamil, PSC 833, benzbromarone, indomethacin, probenecid, agosterol A and analogs, verapamil derivatives, flavonoids derivatives (genistein and flavopiridol), raloxifene-based inhibitors (LY117018, LY329146 and indomethacin), piperazine and piperidine-based compounds as dual MRP1/P-gp inhibitors (*N*,*N*-disubstituted piperazines), isoxazole-based compounds (LY402913), quinazoline- and quinaxoline-derived molecules [5,10,99]	Dexamethasone [248] Rifampicin [248,249] Sulindac [53] TBHQ and quercetin [250] Vinblastine and TBHQ [251]	NR
BCRP (*ABCG2*)	Brain, breast, liver, intestine, placenta and cancer cells [1]	**Anticancer drugs** (anthracyclines (doxorubicin, daunorubicin), epipodophyllotoxins (etoposide, teniposide), camptothecins (topotecan, methotrexate, irinotecan, diflomotecan), anthracenes (mitoxantrone, bisantrene)) **Anti-hypertensives** (prazosin) **Porphyrins** (pheophorbide a, protoporphyrin IX, hematoporphyrin) **Tyrosine kinase inhibitors** (imatinib mesylate, gefitinib, tandutinib, lapatinib) **Natural products** (flavonoids (quercetin, genestein), curcuminoids, phytoestrogens) **Carcinogens** (aflatoxin B, PhiP) **Toxins** (fumitremorgin C, Ko143) **HMG CoA reductase inhibitors** (rosuvastatin, pravastatin, cerivastatin, pitavastatin, atorvastatin, rosuvastatin, simvastatin, fluvastatin) **Metabolite conjugates** (acetaminophen sulfate, estrone-3-sulfate, dehydroepiandrosterone sulfate, estradiol-17-β-d-glucuronide, dinitrophenyl-*S*-glutathione) **Antihypertensives** (reserpine) **Antibiotics** (ciprofloxacin, norfloxacin) **Fluorescent compounds** (Hoechst 33342, BODIPY-prazosin, rhodamine 123) **Antiviral drugs** (zidovudine, lamivudine) [5,10,14,99,174]	***ABCG2*-specific inhibitors** (fumitremorgin C and analogues (Ko143, Ko132, Ko134, Cl1033), eltrombopag) **Compounds also inhibit P-gp and/or MRP1** (elacridar, atazanavir, ritonavir, tariquidar, cyclosporine) **Natural products** (flavonoids, tryprostatin A and novobiocin) **Tyrosine kinase inhibitors** (gefitinib and imatinib) [10,23,99,252]	Efavirenz [10] Quercetin and naringin [253] Oxotremorine, Phenobarbital, Cetylpyridinium chloride, Promazine, Benzetimide, Ifenprodil, Etoposide, Perphenazine and venlafaxine [254] phenobarbital, rifampicin, and omeprazole [255] benzopyrene conjugates [256] Imatinib [257] 3-methylcholanthrene (3MC), troglitazone, resveratrol and omeprazole [258] TBHQ, dibenzoylmethane, quercetin, chrysin, flavone, indole-3-carbinol, Curcumin and resveratrol [259]	NR

P-gp, P-glycoprotein; CITCO, 6-(4-chlorophenyl)-imidazo[2,1-*b*][1,3]thiazole-5-carbaldehyde *O*-(3,4-dichlorobenzyl)oxime; NR, Not reported; TBHQ, tert-butyl hydroquinone.

**Table 2:** Main advantages versus disadvantages of the described in vitro and ex vivo assays (adapted from [1]).

		Advantages	Disadvantages
**In vitro assays**	**Cell-based assays**	Allows to screen for P-gp inducers, activators, inhibitors and substrates. Cell-based transport assays are a classic assay to determine substrates or inhibitors and, more recently, activators. The screening of inducers is also possible but requires the cells pre-exposure to the potential inducer prior to the accumulation/efflux or transport assays, to ensure the de novo synthesis of the transporter. However, to note that an increased expression of a given transporter may not necessarily result in an increase in its transport activity. May provide more information on the interaction between xenobiotics and transporters, due to the intact cell structure. Most of the cell-based assays are functional transporter assays. Can be employed to assess kinetic parameters, such as the half maximal inhibitory concentration (IC_50_) for inhibitors. Can be easily adapted to a high throughput mode (with automation and cell culture in multi-well plates). Additional information may be obtained, such as information on the xenobiotic permeability and transporter localization in cells.	It is more difficult to characterize the xenobiotic effects on one specific efflux transporter, given the expression of multiple transporters in a particular cell line (including cell lines that have been engineered to express a given transporter). The transporters expression levels can change according to the cell culture conditions and number of passages in culture. The cells need to be maintained under aseptic cell culture conditions prior to use. Cell culture media can be expensive, according to the specific supplementation requirements of a given cell line. These assays are more laborious and time consuming than the ATPase assay and membrane vesicular transport studies. Potential underestimation of xenobiotics with low permeability, especially in the accumulation/efflux assays. Potential false negative results in the accumulation/efflux assays for xenobiotics with high passive permeability, given the less relative contribution of a transporter. In the transport assays, polarized epithelium cells with well-defined tight junctions are needed. In the particular case of Caco-2 cells, the development of a proper polarized cell monolayer requires a long-time culture and the cells have multiple efflux transporters expressed. False negative results can be obtained in the transport assays for xenobiotic with high passive diffusion.
	**Membrane-based assays**	Allows to screen for P-gp activators, inhibitors and substrates. Allows to characterize the xenobiotic effects on one specific efflux transporter. Easily employed in a high throughput mode. Easy to maintain the membranes after preparation. Easy to perform. ATPase assays can be used as a high throughput screening tool to identify ligands for ABC transporters—a positive result (either stimulation or inhibition) indicates that the test xenobiotic is a ligand for a specific efflux pump. The membrane vesicular transport assays, contrarily to the ATPase assays, are functional assays and, thus, can be used to distinguish a transporter inhibitor from a substrate. Membrane vesicular transport assays are an effective model for kinetic studies.	Do not allow to screen for P-gp inducers, since de novo synthesis of these proteins cannot be detected. ATPase assays are not functional assays and cannot be used to distinguish between substrates and inhibitors. High intra- and inter-assay variability can occur, particularly in the ATPase assays. In the ATPase assays, the xenobiotics effects should be evaluated at several concentrations to avoid false negative results, since the stimulation or inhibition can occur at either low or high concentrations. False negative results may also be observed for low affinity ligands, since the concentration tested can be limited by the xenobiotic solubility. Membrane-based assays aiming the evaluation of membrane vesicular transport mediated by a given transporter may also give false negative results for lipophilic xenobiotics, which have high nonspecific binding and high passive diffusion.
**Ex vivo assays**		A more accurate determination of the transporter functions in absorption, biliary elimination, renal excretion and brain penetration can be obtained by using isolated perfused intestine, liver, kidney or brain. Allows the evaluation of the potential interplay between transporters and metabolic enzymes. The use of a perfused organ assay allows a much simpler understanding of the role of a transporter in a given organ, when compared with the use of the whole animal, since the concentration of the drug in the target organ can be controlled and the effect from other organs can be avoided.	It is more difficult to characterize the xenobiotic effects on one specific efflux transporter. Need for specific surgical skills and sophisticated equipment. The organ integrity and enzyme activity may become fragile and compromised during long-term perfusions. These assays are more laborious and time consuming. Important to evaluate the potential interspecies differences in transporters when extrapolating data from animal to humans.

## Abbreviations

AB	amyloid-β peptide
ABC	adenosine triphosphate-binding cassette
AD	Alzheimer’s disease
ADMET	absorption, distribution, metabolism, elimination, transport
ADP	adenosine diphosphate
APP	amyloid precursor protein
ASBT	apical sodium–bile acid transporter
ATP	adenosine triphosphate
BBB	blood-brain barrier
BCRP	breast cancer resistance protein
BCSFB	blood-cerebrospinal fluid barrier
BSEP	bile salt export pump
Calcein-AM	calcein acetoxymethyl ester
CAR	constitutive androstane receptor
CD	cluster of differentiation
CNS	central nervous system
Ct	cycle threshold
CYP	cytochrome
DMSO	dimethyl sulfoxide
EC_50_	half maximal effective concentration
EE	ethynylestradiol
ENT	equilibrative nucleoside transporter
FDA	food and drug administration
FRET	Förster resonance energy transfer
FXR	farnesoid X receptor
GAPDH	glyceraldehyde 3-phosphate dehydrogenase
GNT	genistein
h	hours
hAECs	human aortic endothelial cells
hCMEC/D3	human brain endothelial capillary cells
HK-2	human kidney-2 cells
HMG-CoA	3-hydroxy-3-methylglutaryl-coenzyme A
hMVECs	human microvascular endothelial cells
HPLC	high performance liquid chromatography
HPT	intestinal peptide-associated transporter
HPV	human papilloma virus
hTERT IC_50_	human telomerase reverse transcriptase half maximal inhibitory concentration
i.p.	intraperitoneally
K_i_	inhibitory constant
K_m_	affinity constant
LC	liquid chromatography
LLC-PK1	Lilly Laboratories cells - porcine kidney 1
LRP1	low-density lipoprotein receptor-related protein-1
LXR	liver X receptor
MATE	multidrug and toxin extrusion
MCT	monocarboxylic acid transporter
MDCKII	Madin-Darby canine kidney strain II cell line
mRNA	messenger RNA
MRP	multidrug resistance-associated protein
MS	mass spectrometry
NBD	nucleotide-binding domain
NF-κB	nuclear factor-κB
NF-Y	nuclear factor Y
Nrf2	nuclear factor erythroid-derived 2-related factor
NTCP	sodium-dependent taurocholate co-transporting polypeptide
OAT	organic anion transporter
OATP	organic anion-transporting polypeptide
OCT	organic cation transporter
OST	organic solute transporter
P_app_	apparent permeability coefficient
PCR	polymerase chain reaction
PD	Parkinson’s disease
PDB	protein data bank
PEPT	peptide transporter
P-gp	P-glycoprotein
P_i_	inorganic phosphate
PPAR	peroxisome proliferator-activated receptors
PQ	paraquat
PXR	pregnane X receptor
QSAR	quantitative structure-activity relationship
RAGE	receptor for advanced glycation end products
RedRif	reduced derivative
RT-PCR	reverse transcription-polymerase chain reaction
SLC	solute carrier
SV40	simian vacuolating virus 40
TEER	transendothelial electrical resistance
TMD	transmembrane domain
TMH	transmembrane-spanning α-helices
TXs	thioxanthones
URAT	urate transporter
V_i_	inorganic vanadate
V_max_	maximal velocity
Xs	dihydroxylated xanthones
YB-1	Y-box binding protein-1
